# The Role of Natural Killer Cells in Autoimmune Diseases

**DOI:** 10.3389/fimmu.2021.622306

**Published:** 2021-02-25

**Authors:** Umut Can Kucuksezer, Esin Aktas Cetin, Fehim Esen, Ilhan Tahrali, Nilgun Akdeniz, Metin Yusuf Gelmez, Gunnur Deniz

**Affiliations:** ^1^ Department of Immunology, Aziz Sancar Institute of Experimental Medicine, Istanbul University, Istanbul, Turkey; ^2^ Department of Ophthalmology, Medical Faculty, Istanbul Medeniyet University, Istanbul, Turkey

**Keywords:** autoimmunity, cytokines, cytotoxicity, innate immunity, natural killer cells

## Abstract

Natural killer (NK) cells, the large granular lymphocytes differentiated from the common lymphoid progenitors, were discovered in early 1970’s. They are members of innate immunity and were initially defined by their strong cytotoxicity against virus-infected cells and by their important effector functions in anti-tumoral immune responses. Nowadays, NK cells are classified among the recently discovered innate lymphoid cell subsets and have capacity to influence both innate and adaptive immune responses. Therefore, they can be considered as innate immune cells that stands between the innate and adaptive arms of immunity. NK cells don’t express T or B cell receptors and are recognized by absence of CD3. There are two major subgroups of NK cells according to their differential expression of CD16 and CD56. While CD16^+^CD56^dim^ subset is best-known by their cytotoxic functions, CD16^-^CD56^bright^ NK cell subset produces a bunch of cytokines comparable to CD4^+^ T helper cell subsets. Another subset of NK cells with production of interleukin (IL)-10 was named as NK regulatory cells, which has suppressive properties and could take part in immune-regulatory responses. Activation of NK cells is determined by a delicate balance of cell-surface receptors that have either activating or inhibitory properties. On the other hand, a variety of cytokines including IL-2, IL-12, IL-15, and IL-18 influence NK cell activity. NK-derived cytokines and their cytotoxic functions through induction of apoptosis take part in regulation of the immune responses and could contribute to the pathogenesis of many immune mediated diseases including ankylosing spondylitis, Behçet’s disease, multiple sclerosis, rheumatoid arthritis, psoriasis, systemic lupus erythematosus and type-1 diabetes. Dysregulation of NK cells in autoimmune disorders may occur through multiple mechanisms. Thanks to the rapid developments in biotechnology, progressive research in immunology enables better characterization of cells and their delicate roles in the complex network of immunity. As NK cells stand in between innate and adaptive arms of immunity and “bridge” them, their contribution in inflammation and immune regulation deserves intense investigations. Better understanding of NK-cell biology and their contribution in both exacerbation and regulation of inflammatory disorders is a requisite for possible utilization of these multi-faceted cells in novel therapeutic interventions.

## Introductıon

The immune system, formed by a delicate network of cells and effector molecules, aims to provide defense against invaders while protecting the integrity and restoration of the “self”. Innate and adaptive arms of immunity have to work in a harmony. Natural killer (NK) cells, the large granular lymphocytes differentiated from the common lymphoid progenitors, were discovered in early 1970’s (1, 2). They are among a heterogeneous group of innate lymphocytes which have capacity to bridge innate and adaptive arms of immunity (3). These cells are best known for their cytotoxicity against virus-infected or transformed (cancerous) cells, along with their prominent cytokine production capabilities (4, 5). Since their discovery, many studies were performed to better understand the biology of NK cells and their contribution in the delicate network of immunity. NK cells have distinct subsets with disparate developmental origins, repertoires, locations and functions. NK cells could possess natural cytotoxicity, antibody-dependent cellular cytotoxicity (ADCC) and also produce a plethora of cytokines which are similar to that of the well-known CD4^+^ T helper (Th) cell subsets such as Th1, Th2 and Th17 (6, 7). Novel research has attributed a regulatory role for NK cells (8). Both thanks to their production of interleukin (IL)-10 under certain circumstances and also due to being licensed to kill self-reactive cells, these innate cells contribute in maintenance of the immune homeostasis.

Contribution of NK cells in autoimmune and auto-inflammatory disorders has been questioned for decades. Studies proposed important roles for NK cells both for initiation, progression and resolution of these disorders (9). NK cell cytotoxicity as well as NK-derived cytokines take part in the regulation of the immune responses and can contribute to the pathogenesis of many immune mediated diseases including ankylosing spondylitis (AS), Behçet’s disease (BD), multiple sclerosis (MS), rheumatoid arthritis (RA), psoriasis, systemic lupus erythematosus (SLE) and type-1 diabetes (T1D). Some of these diseases are associated with certain genes that act as ligands for NK cell receptors and have the potential to affect NK cell functions (10). While, a dysfunction in regulatory properties of NK cells could end up with failed control of T cell responses and thereby contribute to pathogenesis of certain disorders (11). On the other hand, type-1 interferon molecules; an important component of anti-viral innate responses are closely associated with NK cell functions (12, 13). As NK cells form a bridge between innate and adaptive arms of immunity, their contribution in inflammation and immune regulation deserves intense investigations. Although great knowledge has been accumulated since the initial definition of these cells, better understanding of NK cell biology and their contribution in inflammatory disorders is a requisite to enable utilization of these multi-faceted cells in novel therapeutic interventions.

This review aims to summarize the most up to date knowledge related with the biology and functions of NK cells with a special focus on auto-immune disorders.

## Long-Standıng Members of the Recently Dıscovered Innate Lymphoıd Cells: Natural Kıller Cells

NK cells are accepted as the members of the innate lymphoid cells (ILCs), the recently revealed lymphoid-derived cells of the innate immunity that have vital effector and regulatory functions in immune responses, inflammation as well as tissue regeneration (14). Unlike T and B lymphocytes, antigen receptors of ILCs do not undergo somatic recombination. ILCs are identified with their surface molecules and are negative for lineage markers: CD3, CD4, CD8, CD16, CD34, TCRαβ, TCRγδ, CD19, FcϵR1, CD1a, CD11c, CD94, CD123, CD303, and positive for CD45, CD127, and CD161 (15). ILCs are divided into 3 groups according to the expressed transcription factors needed for their functional development and the cytokines they produce; group 1, group 2, and group 3 ILCs (**Table 1**) (16).

**Table 1:** Innate lymphoid cell subsets and their contributions in inflammatory disorders.

	ILC1	ILC2	ILC3
**Transcription factor**	T-bet	GATA-3	RORγt
**Activated cytokines**	IL-12, IL-15, IL,18	IL-25, IL-33	IL-1β, IL-23
**Mediators produced**	IFN-γ, TNF-α	IL-4, IL-5, IL-13	IL-17, IL-22, GM-CSF
**Function**	Inflammation	Immunity to helminths	Lymphoid tissue development, Intestinal homeostasis, Immunity to extracellular bacteria
**Disease Associations**	Inflammatory conditions, Inflammatory bowel disease	Allergy and asthma	Inflammatory bowel disease

The first identified subset of the ILC group originating from the common lymphoid progenitor (CLP) is the classical NK cell. Group 1 ILCs include both cytotoxic NK cells and non-cytotoxic type 1 (ILC1) cells (16). NK cells respond to tumor cells and intracellular pathogens by releasing cytokines and possessing cytotoxic activity and can also play roles in regulation of the adaptive immune responses. ILC1 subset could be characterized as Lineage^-^, CD45^+^CD127^+^CD161^+^c-kit^-^ and prostaglandin (PDG2) receptor (CRTH2)^-^ (15). T-bet is the main transcription factor for development and function. They are non-cytotoxic cells and can produce interferon-gamma (IFN-γ) that plays a role in the initial response to infections caused by viruses and bacteria. The role of ILC1 group has been demonstrated in the pathology of various diseases such as inflammatory bowel disease, infectious colitis, and diabetes (17).

Group 2 ILCs (ILC2) were first discovered in mouse as natural lymphocytes that produce IL-13 in different tissues, consequently revealed to be present in human intestinal, nasal and adipose tissue, peripheral blood and lungs (18). GATA-3 is the main transcription factor for ILC2 subset and activated by IL-25 and IL-33 cytokines can in turn produce IL-4, IL-5, IL-9, and IL-13. ILC2 subset have a role in response to helminth infections, tissue repair and allergic diseases (19).

Group 3 ILCs (ILC3) are Lineage^-^CD45^+^CD127^+^CD161^+^c-kit^+^ and CRTH2^-^ cells (15), and have 3 different subsets. The first identified cells are lymphoid tissue inducer (LTi) cells that are involved in the formation of secondary lymphoid tissues by secreting lymphotoxin (20). NKp44^+^ ILC3 and NKp44^-^ ILC3 subsets are activated by IL-1β and IL-23, secrete IL-17 and IL-22 and play roles in response to extracellular pathogens, repair of skin tissue, and some autoimmune disease pathologies by assisting neutrophil infiltration. RORγt is the main transcription factor for ILC3 (14, 20).

There is a strong similarity in terms of developmental lineage and functions between ILCs and T lymphocytes. In general ILC subsets are the counterparts of CD4^+^ T cells, and NK cells are the counterpart of CD8^+^ T cells (17).

Briefly, ILCs are the most recently categorized subsets of innate lymphocytes which also encompass NK cells. The identification of various role of ILC subsets showed that there is an important function for ILCs in triggering immunity, inflammation and tissue repair.

## Natural Killer Maturation, Phenotypes, Distribution and Functions

### Natural Killer Cell Development and Maturation

NK cells are developed from common lymphoid progenitors (CLPs) exclusively in bone marrow during adult life but evidence accumulated both from human and mouse studies suggests that precursor and immature NK (iNK) cells can also migrate to secondary lymphoid tissues (SLTs) which include spleen, tonsils and lymph nodes where they undergo terminal maturation and then enter the circulation (21–24). The initial step of this multiple-step process of NK cell differentiation relies on commitment of human stem cells towards the lymphoid/myeloid lineage instead of erythroid/megakaryocyte lineage. During differentiation of human NK cells, Lin^-^CD34^+^CD133^+^CD244^+^ hematopoietic stem cells progress to CLPs following expression of CD45RA molecule to become lymphoid-primed multi-potent progenitors (LMPP). LMPPs are converted into CLPs by expressing CD38, CD7, CD10, and the cytokine receptor CD127 (IL-7 receptor alpha). CLPs have the ability to differentiate into B, T and NK cell progenitors and also to other lymphoid cells (25). CD122 (IL-2Rβ) expression marks an inevitable fate choice in the NK lineage commitment of CLPs (26). NK cell differentiation requires cell-to-cell interactions with stromal cells, the existence of the stem cell factor (SCF), ligand for the fms-like tyrosine kinase 3 (FLT3-L) and IL-7 for lymphoid commitment (27). NK cells are classified according to their sequential stages of maturation which could be designated as NKPs (stage 1), pre-NK (stage 2) and iNK cells (stage 3) (22, 24).

The CD34^+^CD45RA^+^CD39^+^CD10^+^CD123^-^CD127^-^ phenotype is defined by pre-NK cells (pre-NKP_S_) (25). However both NKPs and pre-NK cells have the ability to transform into other types of cells (T cells, dendritic cells (DCs) and other ILCs) by maintenance of CD34 expression (24, 28). NKPs are best characterized with down-regulation of CD34 and by the acquisition of CD122, which is the beta chain of the IL-2 receptor shared by both IL-2 and IL-15 receptors. IL-15 is vital for NK cell maturation, differentiation and survival. Expression of CD122 indicates the one-way fate decision of CLPs into NK lineage (29–31). Meanwhile, mature NK cells gain self-tolerance and effector-functions by loss of CD34 and c-kit accompanied by sequential expression of CD56, CD94, and the killer C-type lectin receptor (CD161) (24, 32, 33). Mature NK cells can progress into 2 final developmental stages, with respect to the expression of CD56 and CD16 (34–38).

**Figure 1:**
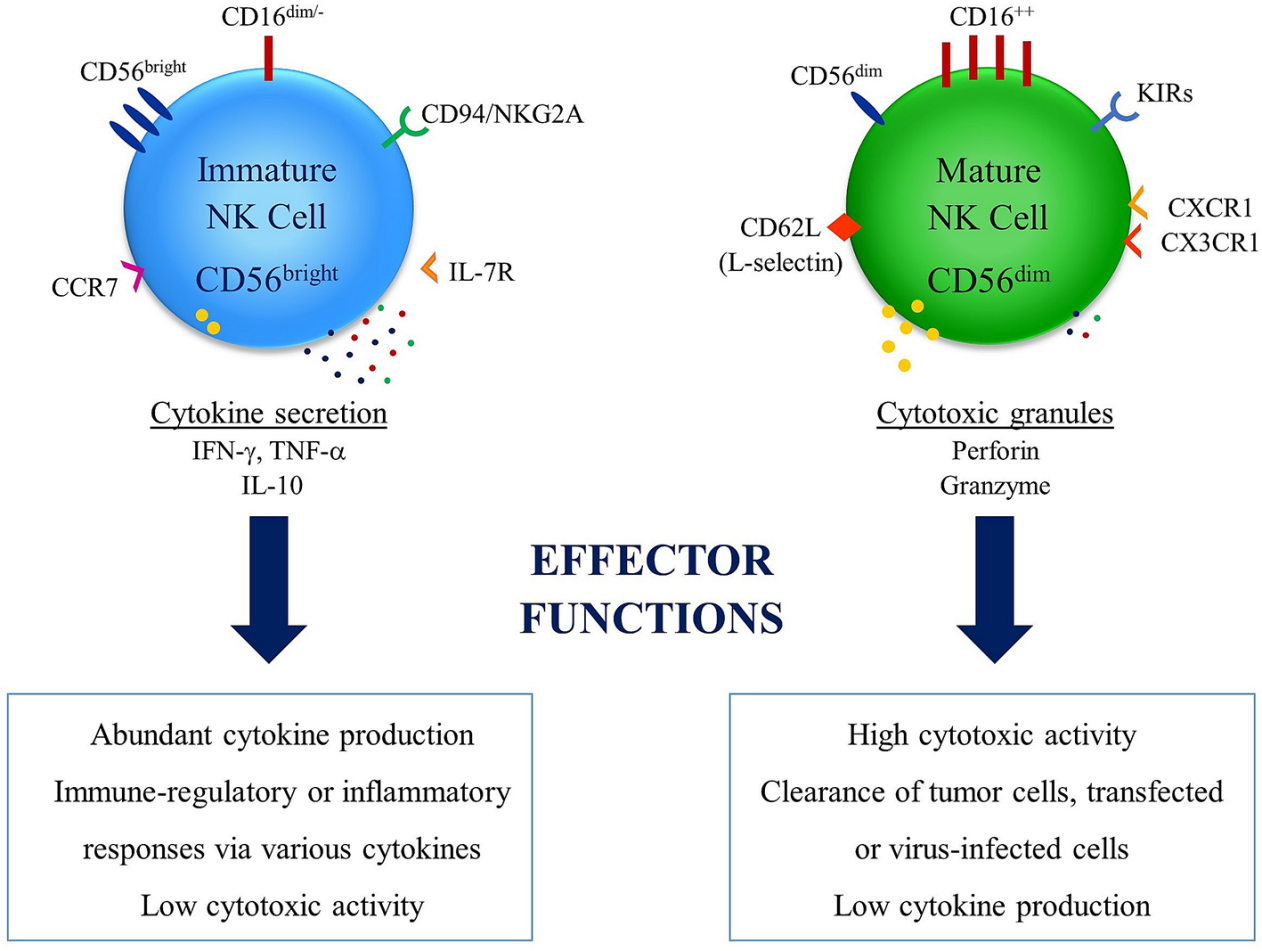
Differential effector functions of NK cell subsets: NK cells have two major subsets in relation with their CD56 expression levels. High expression levels of CD56 is observed in immature NK cell subset, which does not express high levels of CD16 and best known with abundant expression of cytokines. This subset has inflammatory and/or immune regulatory properties and low cytotoxic activity. On the other hand, the subset with low expression levels of CD56 in known as mature NK cell subset, which express high levels of CD16 and is associated with clearance of tumor cells and transfected or virus-infected cells thanks to cytotoxic activity. This subset is also associated with diminished cytokine production capacity.

CD34^-^LFA1^low^NKp46^dim^NKG2D^dim^CD94^-^ subset was defined as immature stage 3a human lymph node NK cells. During NK differentiation, LFA-1 might be upregulated later than CD94. The time series of CD161, NKG2D and NKp46 makes the division of stage 3 into sub-stages 3a and 3b. The cell phase in which CD56 and CD94 were co-expressed at a low density potentially defines an intermediate stage 3b. LFA-1^low^CD56^dim^CD94^dim^CD16^-^ NK cell line was particularly fractional to the Lin^-^CD34^-^LFA-1^low^ cell. CD56^bright^ NK cells may derive from CD161^+^NKG2D^dim^NKp46^dim^CD56^-^ cells which go through the cell process CD56^dim^CD94^dim^CD16^-^ (37). The CD56^bright^CD16^−^ NK cells, which are involved in 4 developmental stages, have immune regulatory and cytokine-producing capabilities and are best recognized with CD34^-^CD117^low^CD94^+^CD16^-^ phenotype. Two different stages (4a and 4b) were described according to NKp80 expression in NK cells at the stage 4 (39, 40). NKp80^-^CD56^bright^ NK cells at stage 4a lack effector functions and best characterized by high expression of NKG2D, NKp30, and NKp46, CD94/NKG2A, CD161. On the contrary, NKp80^+^CD56^bright^ NK cells at stage 4b can produce IFN-*γ* and facilitate perforin-dependent cytotoxicity (22). The final terminally differentiated NK subset gradually gains expressions of CD16, killer Ig-like receptor (KIR) and cytotoxic granules, bringing out a transient population of CD16^+^CD56^bright^ NK cells (41). The final maturation of human NK cells is accompanied by CD56^bright^ NK cells expression loss of CD117, CD127, and CD94/NKG2A receptor, while gaining of CD94/NKG2C and down-regulation of CD56 (42, 43). By downregulation of CD56 expression to become CD56^dim^. The majority of human NK cells in the peripheral blood is the subset which is best characterized by diminished expression of CD56 and express high levels of CD16 (**Figure 1**) (44, 45). Several recent studies have suggested that unlike this traditional model, CD16^+^CD56^dim^ and CD16^-^CD56^bright^ NK cells may also originate from separate lineages (36–38, 45, 46).

### Natural Killer Cell Receptors

Functions of NK cells are regulated by a delicate balance of a number of cell-surface expressed activating and inhibitory receptors. NK cell inhibitory receptors that are specific for human leukocyte antigen **(**HLA)-class I surface molecules of healthy cells aim to prevent NK cell-mediated attack. These could be clarified as two diverse classes of HLA-class I specific inhibitory receptors: the members of the KIR/CD158 family and the CD94/NKG2A (CD94/CD159a) heterodimer (47, 48). Both of the inhibitory receptor types include immunoreceptor tyrosine-based inhibitory motif (ITIM) in the cytoplasmic tail to generate blocking signals. There are also activating forms of KIRs (49) which do not contain cytoplasmic ITIM motifs in their cytoplasmic tail and possess charged transmembrane residues required for association with immunoreceptor tyrosine-based activation motif (ITAM)-bearing molecule KARAP/DAP12 (50). Inhibitory KIRs possess long cytoplasmic tails (L), while activating KIRs utilize short cytoplasmic tails (S) (51). KIR/HLA combinations with high levels of polymorphisms were reported to be linked with either protection from or predisposition to inflammatory, autoimmune as well as reproductive disorders (51–54). Other HLA-specific inhibitory receptor is exemplified by leukocyte Ig-like receptor (LILR) family. LILRB1 (LIR-1/ILT2/CD85), commonly expressed on NK cells utilize cytoplasmic ITIM and recognize HLA-class I molecules. The cytomegalovirus encoded HLA-class I homolog, UL18, expressed on CMV-infected cells, could play a role in host defense by binding to LILRB1 (55). Similar to NKG2A, another HLA-specific activating receptor, CD94/NKG2C expression is limited to late stages of NK cell maturation, whereas CD94/NKG2A is expressed by immature NK cells (56). NK cells express various receptors that can cause their activation through binding to specific ligands that are present on the surface of infected or transformed cells (5). The natural cytotoxicity receptors (NCRs) are classically germ line-encoded receptors which are recognized as the major NK activating receptors (57) and consist of NKp46 (NCR1, CD335) (58), NKp44 (NCR2, CD336) (59) and NKp30 (NCR3, CD337) (60). Induction of NK cytotoxicity against infected cells and tumors is provided by these molecules. NKp30 and NKp46 receptors are present on almost all resting human NK cells, on the other hand, NKp44 expression is almost limited to CD56^bright^ NK cells, but following activation by cytokines, NKp44 could essentially be expressed by all NK cells (5).

NKG2D is a type II transmembrane and C-type lectin-like receptor with activating properties which is expressed in both NK cells and also in cytotoxic T cells. UL-16 binding proteins (ULBPs) and major histocompatibility complex (MHC)-class I chain-related protein (MIC)-A and B; the HLA-class I structural homologs that are upregulated in infected and tumor cells, form the ligands for NKG2D (61). Tumors could escape from the host immune system by shedding of NKG2D ligands from tumor cells (62, 63). As co-receptors, 2B4 (64), NTB-A (65), DNAM-1 (66), CD59 (67), and NKp80 (40) could boost triggering of NK cells already induced by NCRs or NKG2D. NK cells could also express toll-like receptors (TLRs) that induce potent NK cell activation following interface with bacterial or viral products (68–70). Circulating NK cells express low-affinity receptor for the constant region of IgG termed as CD16, which has capacity to trigger antibody dependent cellular cytotoxicity (ADCC) following recognition of the Fc portion of IgG antibodies that are specific to unhealthy cells (71). Several novel treatment options to enhance anti-tumor immunity have been developed over the last decade. Recently, the utilization of NK cells due to their anti-tumoral properties has gained interest for therapeutic intervention (72–74). NK cells express multiple activating receptors such as CD16, also known as Fc*γ*RIIIA, NKG2D, NKp30, NKp44 and NKp46 cytotoxicity receptors, all of which could be used to trigger anti-tumor immunity (75). The production of tri-functional NK cell engagements (NKCES) targeting two activating receptors, NKp46 and CD16, in NK cells and a tumor antigen in cancer cells has been reported (76). As a consequence, tri-functional NKCEs are a new generation of molecules for battle against cancer.

### Transcriptional Approach to Natural Killer Cells

Profiling of the transcriptome is an important process for understanding cell biology. RNA sequencing (RNA-seq) is a recently developed complete transcriptome sequencing technique that uses deep-sequencing technologies and is a fast and a high-throughput method with high detection sensitivity and capacity, provides a more detailed and quantitative view of gene expression, alternative splicing, and allele-specific expression (77–79).

The transcriptional programs that regulate NK cell development is not yet fully understood. Specifically, the transcriptional activation or repression during NK cell ontology is poorly defined (80). Smith et al. analyzed single-cell RNA-seq of NK cell subsets and compared gene expression in unstimulated and IL-2-activated cells from healthy donors (81). They showed that CD56^bright^ NK cells respond most potently to IL-2 stimulation, and there are expressions of GPR183, IL-7R, LTB, GZMK, CD62L, and CCR7 and high expressions of CD2 and KLRC1 (NKG2A), low expressions of FcγRIIIA (CD16) in this subset (81). According to miRNA transcriptome analysis in murine resting NK cells, more than 300 miRNA is expressed following cytokine activation (82). miR-223 that specifically targets the 3′ untranslated region of murine Granzyme B in resting NK cells, was down-regulated as response to IL-15. There is a heterogeneity in bone marrow and blood NK cell-transcriptome data, functionally matured NK cells with high expression of CX3CR1, HAVCR2 (TIM-3), and ZEB2 represents terminally differentiated status with the unique transcriptional profile to single cell RNA-seq analysis (83). Based on murine transcriptome analysis, bone marrow NK cells are divided into five distinct NK cell clusters, and mTORC2-Akt-FoxO1-T-bet axis is important in regulation of immature NK cell genes during development (80).

Recent studies on NK cell function in autoimmunity focused on RNA-seq methods. Kallionpaa et al. analyzed seven children who developed β-cell autoimmunity (84), and indicated that IL-32 was upregulated before appearance of autoantibodies, which was caused by activated T and NK cells. As a result, IL-32 could have a role in T1D, and may be used for detection of abnormal immune cell function prior to T1D onset (84). Lupus nephritis (LN) is a complication of SLE. Arazi et al. showed that there are two NK cell subsets in LN (85). One of both NK cell expresses lacked CD3E and CD3D combined with expression of CD56 (NCAM1) and DAP12 (TYROBP), as well as high expression of cytotoxic genes including PRF1, GZMB, and GNLY. The other NK cell subset was differed by higher expression of KIT, TCF7, IL7R, and RUNX2, and lower expression of PRF1, GZMB, FcγRIIIA, TBX21, and S1PR5 (85). Single cell RNA-seq analysis in rheumatoid arthritis synovial tissue indicated that T, B and NK cells that are associated with RA disease and CD4^+^ T, B and NK cells likely contribute to RA pathogenesis through expression of signaling molecules and their interactions with immune cells and fibroblast populations (86).

### Effector Functions of Natural Killer Cells

NK cells have two critical effector functions. First, NK cells can directly kill cells undergoing malignant transformation (tumor cells) or cells infected with either viral or other intracellular pathogens (6). The cytolytic function of NK cells is critical for the clearance of unhealthy and dysfunctional cells (87, 88). Second effector function of NK cells is the production of a diversity of cytokines in response to activation receptor triggering and also inflammatory cytokine-induced activation (89, 90). The fundamental mechanisms by which effector functions of NK cells mediate protective immunity are critical components of an immune response.

### The Mechanisms of Natural Killer Cell Cytotoxicity

Different mechanisms of target cell recognition by NK cells and how they are considered suitable for being destroyed have been identified. Following target cell recognition by NK cells, target cell killing is provided through the formation of a lytic immunological synapse (IS) that enables NK-induced target cell death through two key mechanisms (4). The first mechanism is involvement of death receptor activation that are located on the surface of the target cells, which consequently leads to activation of the extrinsic apoptotic pathway (91). In the death receptor (extrinsic) pathway, the binding of death receptors like tumor necrosis factor (TNF)-related apoptosis-inducing ligand receptors (TRAIL-R) and Fas (CD95) which are activated by their natural ligands; Fas ligand (FasL/CD95L) and TRAIL, respectively, direct NK cells (88, 92). The NK cell-derived IFN-γ can induce the manifestation of death receptors on target cells, which in turn activates pro-apoptotic signaling programs (93, 94). These death receptors activate the relevant apoptotic mechanisms which includes initiator caspases-8 and -10 by the cytoplasmic death domain (95, 96). Initiator caspases trigger the formation of the apoptosome as a consequence of mitochondrial damage and cytochrome C release (97). The cell death *via* apoptosis occurs as a result of DNA fragmentation induced by caspase 3-activated DNase activation (98).

NK cells store lytic molecules in cytolytic granules that aim to release apoptotic molecules to the target cell following membrane fusion at the IS (4). These cytolytic granules encompass the pore-forming glycoprotein; perforin (99), granzymes, FasL (CD178), TRAIL (CD253), and granulysin (100). Granzyme B and perforin are critical components of NK cells lytic granules. Following entrance of Granzyme B in the target cell, apoptosis could be induced by both caspase-dependent and caspase-independent mechanisms. The caspase-dependent apoptosis pathway of Granzyme B is activated by the cleavage of the apoptotic initiator caspase-8 as well as caspase-3 (101, 102).

Human NK cells could also kill target cells mainly by a lytic granule-independent mechanism, particularly through FasL, which facilitates killing slowly. The conjugation of NK cells with target cells gradually triggers caspase-8 which leads to extrinsic apoptosis (103). Apoptotic signaling *via* FasL includes the mandatory activation of caspase-8 (104) and can proceed *via* mitochondrial-dependent or independent pathways (105). Mitochondrial pathways include the release of cytochrome c for caspase and apoptosis (106, 107). Lytic granules could induce a switch-like induction of Granzyme-B, leading to quick cytotoxicity following one, long conjugation.

### Cytokine Production of Natural Killer Cells

NK cells are known with their production of a variety of cytokines dependent on the inflammatory micro-environment. While NK cells were originally named after their ability to directly lyse target cells, cytokine release by NK cells is another effector mechanism that has capacity to influence T cell responses (108). The hallmark cytokine of NK cells is IFN-*γ*, which could be produced very quickly (2-4 hours) following activation (109). The production of IFN-*γ* by NK cells is a necessity for driving the differentiation of Th1 cells (110). Type 1 and type 2 cytokines could be produced by NK cells (18, 111, 112). Generation of IL-5 in NK cells could be up-regulated by IL-4 and inhibited by IL-12. NK cells can also adversely impact T cells, especially through the production of IL-10 and TGF-β (113). In a study, a NK cell subset was revealed to secrete IFN-*γ* spontaneously. According to this study, NK cells isolated from peripheral blood samples of poly-allergic individuals could be distinguished into separate cytokine-producing NK1 or NK2 subsets (7). These findings indicate that the NK cell cytokine profile is not stable and could fluctuate between IL-4 and IL-12. The stability of cytokine patterns of primed Th1 and Th2 cells are also being discussed. Like T-cell effectors that have been shown to contribute to inflammatory disease pathogenesis, NK1 and NK2 subsets may be provocative and counter-regulatory in allergic and regular immune responses. Cytokines secreted by NK cells include IFN-γ, TNF, and granulocyte monocyte colony-stimulating factor (GM-CSF), all of which enable activation of T cells and other cellular members of innate immunity including DCs, macrophages and neutrophils (114). NK cells could also produce chemokines such as MIP-1α, XCL1 (lymphotoxin), CCL3, CCL4 (MIP-1β), CCL5 (RANTES), and CXCL8 (IL-8), all of which can contribute in migration of effector cells into inflamed tissues (115). Production of cytolytic molecules as well as inflammatory cytokines are induced by different transcriptional regulators in NK cells. The transcription factor; Tbet is lineage-specific and is activated early in NK cell development (116). Signal transducers and transcription activators (STAT)-4 and STAT5 are triggered following presence of IL-12 and IL-2+IL-15 cytokines, respectively (117). The main transcription factor for IL-12, a cytokine crucial for production of NK-cell derived IFN-γ, is STAT4 (118). STAT5 is another important transcriptional regulator for NK cell development, survival, regulation of maturation, and cytotoxicity, which is activated by other cytokines as IL-2, IL-7 and IL-15 (119). Various cells produce a range of inflammatory mediators to prime and sensitize NK cells, especially DCs play a central role. Cross-talk between NK and DCs is described as a key step for NK cell sensitization (120). DCs produce crucial cytokines such as IL-15, IL-12, IL-23, IL-27 and IL-18 cross-talk with NK cells, which contributes in an ongoing immune response (121). On the other hand, NK cells could produce type-1 IFNs that have capacity to prime DCs (122).

### Natural Killer Cells in Health and Tolerance

Every immune response needs to be regulated to be specific to per individual pathogen and also to prevent subsequent tissue destruction and/or autoimmunity. Induction and maintenance of immune tolerance is a key component of a healthy immune response both to our own flora and to environmental harmless antigens. A defect in tolerance could end up with autoimmune and auto-inflammatory reactions and allergic reactions, while deficient immunity could lead to immune deficiencies and cancer development. Immune tolerance is an active state of immune response with underlying mechanisms which are maintained by a complex network of regulatory cells and cytokines (38, 123).

It is very well known that although being members of innate immunity, NK cells at the interface between innate and adaptive arms of immunity contribute in immune responses both with their cytotoxicity and production of a number of cytokines. Among the NK subsets, CD16^-^CD56^+^ subset is best characterized with high cytokine secretion proficiency, which could act either inflammatory or regulatory (8, 124). A small fraction of NK cells was revealed to limit antigen specific T cell proliferation *in vitro* in IL-10 dependent manner, which was termed as regulatory NK (NKreg) cells (8). A number of studies also proposed regulatory roles for NK cells, either by production of IL-10 or by counter-balancing the ongoing inflammation (125–127).

The anti-inflammatory cytokine IL-10 is a member of type II cytokine family and serves as a potent suppressor of inflammatory and involved in the pathogenesis of various autoimmune diseases. IL-10 is expressed mostly by activated monocytes/macrophages, NK cells, DCs, mast cells, T lymphocytes (mainly Th2 subsets), and B lymphocytes and IL-10 has capacity to inhibit costimulatory molecules expressed on macrophages and also NK cell activation (128). The cytotoxic properties of NK cells also contribute in immune regulation possibly by killing autoreactive cells. NK cells could exploit cytotoxicity against T cells, DCs, in order to limit excessive inflammation during viral infections. CD4^+^ T cell suppression simultaneously limits B cell-driven humoral immunity, all of which acts on the critical balance between immunity vs excessive inflammation with potential to induce tissue damage (129–131). A mouse model in which extreme inflammation was triggered by viral infection revealed worsened disease progression was associated with depletion of NK cells or presence of NK cells which lacks perforin mediated cytotoxicity (132).

### Natural Killer Cells in Autoimmune Diseases

Altered functions and also the regulatory properties of NK cells could be influential in a number of diseases with autoimmune/auto-inflammatory background.

#### Behçet’s Disease

Behçet’s disease is a systemic vasculitis of unknown etiology that can affect many organ systems including but not limited to the skin, eyes, brain, lungs and joints. The exact immunopathogenesis of the disease remains unknown but is believed to be multifactorial by the contribution of genetic susceptibility and environmental factors. HLA-B5/B*51 allele has the strongest genetic association with the disease. Data from genome wide association studies (GWAS) have highlighted some important loci that contribute to the disease susceptibility: *IL10*, *IL23R*, *HLA-A, HLA-B*, *CCR1*, *STAT4*, endoplasmic reticulum amino peptidase 1 (ERAP-1), MEFV and TLR-4 (133–136). Some other class-I MHC alleles (HLA-A*03, -A*26, -B*15, -B*49, -B*27, and -B*57) also contribute to the risk of BD (137). A strong association of SNP, rs1800871 of the IL-10 promoter region was also demonstrated in patients with BD (138).

Immune responses in BD are relatively complex as the disease has both autoimmune and auto inflammatory characteristics (139). The predominant immune response responsible for various organ involvements may change and there might be multiple immunologic pathways responsible in various tissues. Both innate and adaptive immune responses were shown to be associated with the inflammatory attacks and treatment responses in BD (12, 13). Two relatively old studies demonstrated that NK cell numbers increased in peripheral blood in BD, while NK function was decreased in these patients (140, 141). Current studies demonstrated that these changes in NK cells were probably dependent on the organ involvement. BD patients with arthritis had decreased numbers of NK cells in their blood and synovial fluid compared to healthy controls and AS patients (142), while there was no such a change in NK cells and their subsets (CD16^bright^CD56^dim^ and CD16^dim^CD56^bright^ cells) in mucocutaneous BD patients during the active phase of the diseases compared to healthy controls and remission phase (125). Two studies with heterogeneous groups of BD patients demonstrated an increase in the numbers of cytokine secreting NK cells (143, 144), while one of them also demonstrated a decline in the numbers of cytotoxic NK cells (144). Azathioprine-treated patients had a reduced ratio of NK cells compared to healthy individuals while there was no such a change in patients treated with other medications (prednisolone, colchicine, or mycophenolate mofetil). This data suggested that azathioprine could lead a reduction in NK cell numbers in BD (144). In earlier studies, the cytotoxic activity of NK cells were found to be decreased during active disease compared to healthy individuals and patients in remission (140, 145, 146). In BD patients with pulmonary involvement, NK cells isolated from broncho-alveolar lavage fluid had reduced cytotoxic activity compared to the bronchoalveolar lavage fluid of RA patients and healthy individuals (147). In BD patients with mucocutaneous involvement, the cytotoxic activity of the NK cells remained unchanged and there was no significant change in CD107a expression compared to healthy individuals. There was also no significant difference between patients that had frequent oral ulcers and rare oral ulcers (125). In heterogeneous groups of BD patients, CD107a expression of total NK cells were found to be upregulated when compared to healthy individuals (144, 148). No difference of CD107a expression among active and inactive patients was observed. Despite the increase in CD107a expression, perforin and granzyme B production remained unchanged in BD patients compared to healthy individuals (144). Disease activity scores and treatment modalities were also not associated with any change in the cytotoxic activity of NK cells (148).

Cytokine production by NK cells influences both adaptive and innate immune responses. A Th1 dominated adaptive immune response and increased expression of IFN-γ in BD patients was demonstrated (142, 149–151). Cytokines that are associated with Th1 response are also known to activate NK cells and stimulate secretion of IFN-γ by NK cells (152). In BD, IL-12 secreted by Th1 cells activates NK cells, induces their migration to the site of inflammation and contributes to tissue damage in these patients (153). IFN-γ-secreting NK1 cells were increased in BD patients with mucocutaneous involvement, while the number of the other NK subsets (NK2, NK17 cells, and IL-10-secreting regulatory NK cell subsets) are decreased in these patients (125). A similar NK1 predominated cytokine secretion pattern was also observed among CD56^bright^ NK cells in a more heterogeneous group of BD patients which confirms the above-mentioned observation (144). Certain functional changes were observed in NK cells during active and remission phases of BD patients with uveitis. TNF-α, IFN-γ, IL-2 and IL-4 secretion was increased in NK cells during the active inflammation period, while IL-4 and IL-10 producing NK cells increased during the remission phase. This data further supported the idea that Th1/NK1-type immune response was responsible for inflammation in these patients. Th2/NK-2 type immune response is increased during remission (126). IL-10 secreting NK cells were described as a regulatory subgroup of NK cells that takes part in the resolution of inflammation (8) and this regulatory NK cell subgroup was also increased during the remission phase of Behçet’s uveitis (126).

KIR3DL1 is a polymorphic, inhibitory NK cell receptor specific for the Bw4 epitope of class-I MHC molecules (154). The interaction of KIR3DL1 and Bw4 epitope was proposed as one of the mechanisms to explain HLA-B51 and disease association (155). However, neither inhibitory KIR3DL1 nor activating KIR3DS1 alleles were associated with BD among the patients that carry HLA-B*51, HLA-B with a BW-4 motif or not (156). KIR3DL1 expressing NKB1^+^CD56^+^ NK cells were increased among BD patients with uveitis while the expression of the other NK receptors such as CD94 and CD158b remained unchanged in the CD56^+^ NK cells of BD patients (157). Killer cell lectin-like receptor subfamily C, member 4 (KLRC4) belongs to the NKG2 receptor family expressed primarily in NK cells and plays an important role in the regulation of NK functions. A novel locus of KLRC4 gene was found to be associated with BD in a whole-genome screening study with multi-case families (158).

NK gene complex encodes C type lectin receptors CD94 and NKG2D, NKG2F, NKG2E/NKG2C and NKG2A that plays important role for the regulation of the cytotoxic activity of NK cells (159). CD94/NKG2A is an inhibitory receptor and CD94/NKG2C is an activating receptor on NK cells that bind to HLA-E as ligand. Certain alleles of CD94/NKG2A and their ligand HLA-E are associated with an increased risk of BD, while there was only a difference in CD94/NKG2C alleles between patients with joint and eye involvement (160). Two of the NK cell receptor genes; MBL2/rs1800450 and KLRC4/rs2617170 also contribute to disease susceptibility in Chinese Han population (161). In a study designed to evaluate phenotypic changes in NK cells, CD94 expression was increased in CD16^+^CD56^+^ NK cells of BD patients compared to healthy individuals, while KIR3DL1 expression remained unchanged. This study also supported the regulatory role of CD94 in patients with BD (162). In addition, NKG2D expressing NK cells were also increased in patients with BD compared to healthy individuals and there was a positive correlation between the disease activity scores and NKG2D expression of NK cells (148).

Many of the genes associated with BD have close ties to NK cells and their functions. Behçet’s patients tend to form clusters by their organ involvement (such as vascular involvements, acne arthritis enthesitis cluster, parenchymal Neuro-Behçet and ocular BD) (163). It is unclear whether different clusters have predominance of different immunopathogenetic mechanisms. Above-mentioned studies demonstrated a heterogeneity in the NK associated changes in different clusters. In summary, there is an increase in the numbers of cytokine secreting NK cells and these cells are skewed to a NK1 type of immune response especially during the active phase of the disease, while NK2 and NKreg activity is increased during the remission phase of the disease.

#### Multiple Sclerosis

Multiple sclerosis is a chronic and inflammatory disorder of the central nervous system (CNS) where demyelination causes the characteristic axonal/neuronal loss and gliosis (164). In about 85% of patients, the disease presents with a relapsing-remitting (RR) course which occurs with acute attacks and subsequent remissions (165). MS is a multifactorial disease and it is commonly accepted to have an autoimmune etiology with the contribution of environmental factors in individuals with genetic predisposition (166). Autoreactive CD4^+^ T cells specifically targeting the myelin components of the CNS along with a large number of immune cells, have been considered to have crucial functions in triggering inflammatory processes (167) and cause demyelination and neuro-axonal damage (168). The studies based on immunologic mechanisms revealed the involvement of T (especially Th17 and CD8^+^ T cells) and B lymphocytes (169–171) in the pathogenesis of MS. Due to the observation of positive healing effects on MS patients by starting the therapy with daclizumab; a monoclonal antibody that blocks IL-2 receptor alpha chain (IL-2Rα; CD25), NK cells gained importance for MS studies due to having IL-2R (172, 173). However, the function of NK cells seems to be controversial based on the conclusion of the studies demonstrating their both beneficial and deleterious roles in MS and in experimental auto-immune encephalomyelitis (EAE) induced in rodents (174, 175).

NK cells display their activities depending on the net signal obtained from activating and inhibitory receptors (176). Thus, several studies demonstrated the expression profiles of NK cell receptors (NKRs) in MS and investigated the involvement of NKRs in the pathogenesis of the disease.

The first study exploring the function of KIRs and their HLA ligands in MS development revealed a significantly deficient HLA-Bw4 molecule in patients compared to healthy individuals, suggesting a protective role for HLA-Bw4 in MS (177). HLA-Bw4 is the ligand for KIR3DL1, an inhibitory receptor of NK cells, and the engagement of self HLA-Bw4 molecule with KIR3DL1 gives NK cells a funcional proficiency in humans. Therefore, the deficiency of HLA-Bw4 molecule was considered to lead to inadequate responses to infections and increased risk for MS, due to the functional insufficiency of NK cells (176). However, the fact that many allelic variants of KIR3DL1 have different expression patterns and HLA-Bw4-recognition capability requires further investigation of KIR3DL1 and HLA-Bw4 variants. In addition, decreased frequency of the inhibitory KIR2DL1 and its ligand HLA-C2 as well as elevated frequency of activating-receptor KIR2DS4 were detected in MS patients, which demonstrated an activatory profile for NK cells (177). Another study showed KIR2DL2, an inhibitory receptor related with viral infections, was amplified in MS patients that were infected with herpes virus (HSV)-1 (178). In addition, NK cells expressing KIR2DL2 were demonstrated to fail in controlling HSV-1 infection (179). Altogether, these findings demonstrate KIRs and their ligands might have important functional roles in MS, although it is necessary to investigate the functions of their allelic variants in more detail.

CD94:NKG2A complex, which is another inhibitory receptor of NK cells was shown to prevent killing of self-reactive T cells by NK cells, in EAE (180). Activating receptor NKG2D, a molecular stress sensor, is predominantly expressed on NK cells besides CD8^+^ and *γ*δ^+^ T cells and binds its ligands lowly expressed on healthy cells (62). The increase in expression of stress molecules such as MICA/B and ULBP in cancers and viral infections (63, 181) lead to the up-regulation of NKG2D molecules which has a crucial role in immune surveillance (182). In sera of MS patients, MICB levels were demonstrated to be elevated (183) and also MICB*004 allele was shown to be associated with increased susceptibility for MS (184). Moreover, NKG2D^+^ NK cells were shown to lyse activated CD4^+^ T cells with the help of upregulated NKG2D ligands (185, 186). All these findings give a clue that NKG2D expressions of NK cells could be elevated in patients with MS in response to their increased ligands. However, it is known that chronic exposure to NKG2D ligands leads to down-regulation of NKG2D expression resulting in diminished cytotoxicity (187, 188). Conformingly, our previous study has demonstrated NKG2D expressions of CD56^+^ NK cells were decreased in untreated RR-MS patients in comparison with patient group treated with IFN-β and also revealed the elevation in treated group was negatively correlated with their expanded disability status scale (EDSS) scores (189). These findings suggest IFN-β therapy might be releasing the suppression of NKG2D expression on NK cells in RR-MS patients and may prove beneficial *via* providing NK cell activation.

NK cells have the ability of regulating autoimmune mechanisms by cytokine secretion or direct cytotoxic activity on effector cells such as autoreactive T cells or antigen presenting cells (APCs) (190, 191). However, they might also lyse oligodendrocytes, astrocytes and microglia through NKG2D ligands (192, 193) suggesting a double-edged sword function for NK cells in MS. A number of studies demonstrated a deficiency in cytotoxic activity of NK cells in peripheral blood of MS patients (194–196). Moreover, decreased NK cell cytotoxic activity was present in RR-MS patients before the occurrence of CNS lesions and the onset of clinical symptoms, and deficient NK cytotoxic activity was correlated with MS attacks and manifestation of new lesions (195, 197). In EAE, depletion of NK cells (198, 199) or blocking NK cell-homing to the CNS (200) were demonstrated to result in relapses and increased mortality. Insufficient cytotoxic activity of NK cells might lead to an increase in the proportion of autoimmune cells that in turn could be associated with disease progression. On the other hand, NK cell cytotoxicity towards viruses must also be taken into account in the pathogenesis of MS.

Viral infections such as Cytomegalovirus (CMV), Epstein-Barr virus (EBV), and human herpes virus (HHV)-6 have been considered as candidate inducers of MS as a result of molecular mimicry (201, 202). Since IFN-β, being effectively utilized in MS treatment, is an anti-viral cytokine, it supports the hypothesis that viral infections are accepted as risk factors in the development of MS (203). Today, there is evidence for a failure in controlling chronic viral infections is involved in the triggering of autoimmune responses leading to MS (204). Therefore, the cytotoxic activity of NK cells also appears to be vitally important in protecting from the development of MS, as they are the early defenders against viral infections. However, the association between decreased cytotoxicity and MS attacks suggests that the importance of the cytotoxic activity of NK cells is not limited to preventing the disease development *via* controlling viral infections, but also it is beneficial for dampening the disease progression.

CD56^bright^ subset of NK cells has been much explored in the context of MS due to its inflammatory and immune-regulatory functions. CD56^bright^ NK cells mediate T cell responses *via* secreting cytokines like IFN-*γ* and IL-10 (205) or they can suppress T cell proliferation (206, 207). It is known that CD56^bright^ NK cells could also kill certain target cells, especially healthy autologous T cells (208, 209). Indeed, CD56^dim^ and CD56^bright^ NK cells were shown to lyse T cells which are at different activation status, in response to different stimulants (186, 210). Thus, the differences in functional features of CD56^dim^ and CD56^bright^ NK cells require interpretation of the data from studies of NK cells in MS separately.

The quantitative measurements of CD56^bright^ NK cells demonstrated that their frequencies in untreated MS patients are similar to healthy individuals (211, 212). However, several studies have shown that patients with treatment (IFN-β derivatives, daclizumab, and alemtuzumab) had increased levels of CD56^bright^ NK cells compared to healthy individuals (210, 212–214) which was also revealed by a previous work of our group (127). This finding proposes a protective function for CD56^bright^ NK cells in MS, however, functional assays of NK cells are not as clear. The major cytokines secreted from CD56^bright^ NK cells are IFN-γ, IL-10, IL-13, tumor necrosis factor (TNF)-α and granulocyte–macrophage colony-stimulating factor (GM-CSF) that are dependent on the milieu (45). Due to its important roles in autoimmunity, IFN-γ (and IFN-γ producing Th1 cells) was considered as a potential pathogenic factor in MS development (215, 216). Since being one of the main cytokines produced by NK cells, IFN-*γ* levels in NK cells (especially in CD56^bright^ cell subset) of MS patients have been measured in several studies, and shown to be impaired in CD56^bright^ NK cells of untreated RR-MS patients (217). In our study confirming the inadequate IFN-*γ* secretion of NK cells, IFN-*γ* levels of CD56^bright^ cells were also indicated to be down-regulated in treated RR-MS patients as well as untreated patients, even after the addition of IL-12; the main stimulator of IFN-*γ* (127). In concordance with that, we also detected reduced NK cell cytotoxicity in RR-MS patients both with the presence or absence of treatment, revealing that a functional disability of NK cells could be significant in MS pathophysiology.

Although more evidence is required to clearly define their exact role, NK cells seem to be essential in preventing both the development and progression of MS.

#### Systemic Lupus Erythematosus

Systemic lupus erythematosus is a complex systemic autoimmune disorder which is characterized by chronic inflammation resulting in widespread organ dysfunction and various clinical occurrences such as vasculitis, arthritis, nephritis and neuropsychopathy (218, 219). SLE has a relapsing-remitting course and mostly occurs in reproductive-age females. Although the course of the disease in many patients with SLE is mild, it is life-threatening in the rest. Like the other autoimmune diseases, the etiology of SLE is also not clearly defined, however, environmental, immunological and hormonal factors together with genetic predisposition are known to be involved in the etiopathology of this disorder (220). In SLE patients, apoptosis is increased which is thought to be due to the environmental factors such as ultraviolet light, infections and toxins. Insufficient immune clearance of apoptotic cells results in the exposition and accumulation of self-DNA and nuclear antigens (221). These nuclear particles could trigger TLR of APCs (222, 223). As a result of continuous activation of APCs by autoantigens, T cells are activated, proliferated and initiate autoreactive polyclonal B cell activation which is characteristic for SLE (224). The autoantibodies are secreted from B-lymphocytes against nuclear proteins and DNA. The anti-double-stranded DNA (ds-DNA) autoantibodies are specific marker for SLE diagnosis (225) and they form immune complexes and by this way cause tissue damage (226, 227). The accumulation of immune complexes leads to prompt immune system by activating complement system and binding γ receptors (228). Many other immune system members also contribute in disease development (229) and novel evidences point the possible participation of NK cells in SLE.

Although the studies exploring the contribution of NK cells in SLE are not quantitatively comparable with the other autoimmune diseases such as MS or RA, findings demonstrated the numerical decreases in circulating NK cells of SLE patients, associated with the clinical symptoms and disease activity (230–232). This reduction has been linked to the increased serum levels of IFN-alpha (IFN-α) in SLE patients (233). IFN-α; produced in response to viral infections by different cell types [especially by plasmacytoid dendritic cells (pDCs)], is an important cytokine involved in immune regulation (234). The immune complex-mediated IFN-α production by pDCs is characteristic for SLE. There are many reports demonstrating the contribution of IFN-α in the pathogenesis of SLE, though the exact mechanism of IFN-α in which way it affects the disease remains unclear. It has been shown in a study that IFN-α mediates the activation-induced cell death (AICD) so that lead to reduce the frequency of circulating NK cells in SLE (233). Conversely, NK cells could promote the production of IFN-α by pDCs (235, 236). However, the crosstalk between NK cells and pDCs in SLE needs to be much explored.

In addition to the numerical reduction, cytotoxic activities of NK cells have been also demonstrated to be suppressed in SLE (230, 237, 238). The decreased NK cell cytotoxicity has been shown not to correlate with degranulation defects which is detected by CD107a expression (239, 240). Interestingly, the functional insufficiency seen in cytolytic activity is not observed in IFN-*γ* production by NK cells. Indeed, IFN-*γ* production of NK cells in patients with active SLE in response to various stimulants is significantly increased when compared with healthy individuals (239, 241) and the frequency of NK cells producing IFN-*γ* has been shown to correlate with the levels of serum IFN-α (239). In a murine model of the disease, chronic circulation of high levels of IFN-*γ* was demonstrated to trigger a SLE-like syndrome (242), supporting the role of IFN-*γ* as a major effector molecule in the pathogenesis of the disease. Recently, the levels of blood IFN-*γ* has been discovered to correlate positively with anti-ds-DNA levels and SLE activity (243). As a main source of increased IFN-*γ* secretion, NK cells are seemed to have an importance in SLE pathogenesis. Confirming this hypothesis, NK cells were reported to participate in activation and production of IFN-*γ* in an amyloid-induced lupus-like syndrome model suggesting their involvement in the pathogenesis and development of SLE (244).

Because NK cells activities are controlled by the activating and inhibitory receptors, they have been also investigated in the studies of SLE. The presence of auto-antibodies against inhibitory CD94/NKG2A receptor was described in SLE patients (245). In another study of the researchers, anti-KIR autoantibodies were detected in a group of SLE patients. IgG from anti-KIR positive patients were shown to reduce the cytotoxic activities of NK cells suggesting NK cell functions are defective due to autoantibodies toward these receptors (246). The expression levels of the activating receptors NKG2D and DNAM-1 have been revealed to be reduced in patients with active or inactive stage of SLE, compared to healthy individuals (247, 248). Beside of the studies demonstrating NKG2D positive cell frequency was lower in SLE patients, there are also findings indicating that there is no difference between the groups (241, 249). Another study demonstrated activating receptor NKp46 was higher whereas inhibitory receptors KIR2DL3 and KIR3DL1 were diminished in SLE patients in comparison with healthy individuals (241). Also, they were revealed to express increased levels of activating receptor CD69 in patients with active disease (250). The variability in data regarding activating and inhibitory receptor ratios of SLE patients might be due to the clinical manifestation of patients, treatment type or conditions of assays. Moreover, variable results might be gained from NK cell subgroups, having different functional features.

At this point, it is important to investigate NK cells in two main groups which are mainly named as immune-regulatory CD56^bright^ and cytotoxic CD56^dim^ subsets of NK cells. An augmented CD56^bright^ NK cell proportion has been observed in SLE patients, regardless of the disease activity (249). The proportions of CD56^bright^ and CD56^dim^ NK cells were observed to be similar among active and inactive SLE patients as well as healthy individuals while CD56^dim^ NK cells were tended to be decreased (251). Also, it was shown in the same study that the status of decreased CD56^dim^ NK cells was activated and their IFN-*γ* levels were increased. Granzyme B^+^ CD56^bright^ cell ratios were found to be higher in active phase compared to in inactive phase of SLE and those in healthy individuals (252). In addition, TNF-α levels of CD56^dim^ NK cells but not CD56^bright^ NK cells from active SLE patients were demonstrated to be lower than inactive SLE patients and healthy individuals. Although these findings indicate the presence of functional differences between two subgroups of NK cells in SLE, the number of studies on this field is almost negligible.

Based on the findings obtained from existing studies, NK cells seem to involve in the pathogenesis SLE. However, it is unclear if the defects in NK cell functions are the cause or a consequent of the disease process or the treatment. More studies are required to clarify the functions of NK cell contribution to the pathogenesis of SLE and it is necessary to investigate their roles in the disease by dividing NK cells to two separate subsets. Moreover, active and inactive phase of SLE and clinical manifestations should be taken into consideration.

#### Rheumatoid Arthritis

Rheumatoid arthritis is a chronic and multi-systemic disease which presents with the inflammation of synovium leading to progressive cartilage, joint and bone destruction, all of which result in deformity and disability (253). The genetic and environmental factors together with irregular immune responses are known to be the triggers of the disease (254). The immune mechanisms involved in the pathophysiology of RA are not well defined, however, evidences demonstrate the participation of both innate and adaptive immunity.

Osteoclasts play critical roles in pathogenesis by causing periarticular bone loss which is characteristic for RA (255). In healthy individuals, the formation and resorption of bone are controlled by osteoblasts and osteoclasts, respectively. However, this balance has been broken towards to the direction of osteoclastic activity in RA patients (256). TNF-α is a prominent cytokine promoting the osteoclastogenesis in RA. Synovial fibroblasts and macrophages induced by TNF-α secrete IL-1, a pro-inflammatory cytokine, which activates osteoclasts and triggers the inflammatory process (255). Especially after demonstration of decreased inflammation and bone resorption in response to anti-TNF−α treatment (257), TNF-α was accepted to be the master cytokine of the inflammation and progression of RA (258).

In health, the synovial membrane does not contain cells, whereas a bulky cellular infiltration is observed in that of RA patients (259). This inflammatory infiltrate consists of innate and adaptive immune cells (such as T and B lymphocytes or DCs) interacting with each other and leading to disease development. DCs, by triggering the activation of adaptive immune cells, play an important role in RA development (260). NK cells have also been shown to accumulate in the synovial fluid of RA patients (261, 262). However, it has not been well clarified whether the elevation of NK cell levels in the synovium of RA patients is a result or is one of the underlying causes of the disease. In the presence of studies suggesting NK cells as both pathogenic and protective players in RA, they are considered to be involved in the pathogenesis. A protective role for NK cells was suggested in an animal model of collagen-induced arthritis (CIA) by the finding that antibody-mediated NK cell depletion led to aggravation of the disease (263). However, another study presented an opposite result through this method by demonstrating the healing of CIA and counterwork of bone loss upon NK cell depletion (264).

Despite their increased ratios in inflamed synovium, NK cells have been shown to be decreased in peripheral blood samples of RA patients when compared with that of healthy subjects (265, 266). Additionally, it has been demonstrated in a study that activated NK cells were decreased in peripheral blood of RA patients compared to healthy subjects whereas the frequency of resting NK cells was not altered (267). These findings suggest that activated NK cells might be infiltrated to the inflamed synovium of RA patients. It is important to know the function of NK cells which are abundant in the synovium. The contribution of NK cells in the disease has been thought to be *via* inflammatory cytokine secretion or by interaction with the other members of the immune system involved in the pathogenesis. NK cells obtained from the synovial fluid of patients with RA and co-cultured with monocytes were demonstrated to prompt monocyte differentiation into osteoclasts (264).

As it is known, NK cells might act as protective players by communicating with the other immune cells *via* cytokine secretion or by lysing autoreactive immune cells which cause autoimmune disorders. On the other hand, NK cells might produce inflammatory cytokines or directly have a pathogenic function in autoimmunity *via* cytotoxic effects. Thus, examining CD56^dim^ and CD56^bright^ NK cell subsets which have functionally differences, separately, might guide us to define the role of NK cells in RA. CD56^bright^ NK cells with prominent cytokine secretion have been shown to be significantly increased in inflamed synovium of RA patients (268). It has been hypothesized that CD56^bright^ NK cells in the synovium could abundantly produce pro-inflammatory cytokines such as TNF-α or IFN-*γ* (259). Actually, it is known that by producing TNF-α and IFN-*γ* and by direct contact, activated NK cells can induce the maturation of DCs which consecutively activate NK cells as well as T and B lymphocytes (269, 270).

TNF-α; being the conductor cytokine of RA, is the most studied cytokine in functional analyses of NK cells in RA pathophysiology and has been shown to regulate the differentiation and induce the maturation of NK cells (271). In recent studies, a pathogenic role of Th17 cells has been demonstrated in autoimmune syndromes containing RA and shown to be elevated in the peripheral blood samples and synovial fluids of patients in active phase of disease (272). It has been propounded that NK cells could prevent autoimmune responses by secreting IFN-*γ*, which inhibits Th17 cell differentiation from precursor cells (273) and suppresses the osteoclastogenesis (274). A study showing elevated levels of TNF-α of NK cells in RA patients compared to healthy subjects demonstrated that IFN-*γ* secretion was also tended to be increased (275).

Besides producing cytokines, NK cells have been considered to have importance in regulating autoimmune disorders by killing autoreactive immune cells. It was found that the cytotoxic activity of NK cells was decreased in RA patients in comparison with healthy subjects (276). Additionally, another study established the frequency of perforin-positive NK cells was reduced in patients with RA (275).

Consequently, these findings suggest a functional impairment for NK cells in RA pathophysiology, at least in their cytolytic functions. However, the studies of NK cells in RA patients are nominal to understand their involvement in the pathogenesis. Therefore, the exact role of NK cells in the RA needs to be elucidated.

#### Ankylosing Spondylitis

Ankylosing spondylitis is a seronegative, immune-mediated rheumatic disease associated with joint inflammation and extra-articular manifestations such as uveitis, enteritis, and gut inflammation (277). The strongest genetic risk factor for AS patients is the carriage of HLA-B27, a MHC-class I molecule (278, 279). Certain polymorphisms of the endoplasmic reticulum-associated aminopeptidase 1 (ERAP1) gene have also a strong association with AS, which selectively affects individuals with HLA-B27–positivity (280). NKG2D expression of NK cells was reduced in transgenic mouse which highly expresses ERAP-1, in comparison with the wild type and the other transgenic mouse with low ERAP-1 expression (281). These results indicated that disease-associated ERAP-1 variants could be influential on NK functions. Other genetic associations of AS include certain KIR genes in different populations (KIR2DL1, KIR3DL1, KIR2DS5, KIR3DS1 and KIR2DL5) (10, 282–285). Increased expression of KIR3DL2 was found in the T and NK cells of AS patients (286, 287), but there was no difference in the expression of KIR3DL1 between AS patients and controls (286). In addition, HLA-B27 binding to KIR3DL1 could initiate inhibitory signals to NK cells (288). The presence of certain peptides (such as certain EBV epitopes) in the peptide binding groove of HLA-B27 could limit its ability to bind KIR3DL1 and thereby influence NK functions (289, 290). Therefore, peptide specificity of HLA-B27 molecule, trimming of the peptides by ERAP-1 and the function of KIR molecules can potentially influence NK function through the above-mentioned pathways.

There is conflicting data regarding the changes in the peripheral blood proportions of NK cells in patients with AS. Although two studies demonstrated an increase in the number of NK cells in the peripheral blood of AS patients (291, 292), this observation could not be replicated in another study (230). There was also a prominent increase in the expression of carcino-embryonic antigen-cell adhesion molecule (CEACAM1) expression on NK cells in peripheral blood of AS patients and CEACAM1 was able to inhibit NK cytotoxicity *in vitro* (292). The functional subsets of NK cells were further investigated in AS and the cytotoxic CD16^+^ CD56^dim^ subset of NK cells were increased in comparison with healthy individuals (293). A20 functions as an inhibitor of inflammatory cytokines in several cell types and A20 expression was decreased in the CD56^bright^ NK cell subset of AS patients compared to healthy individuals (294). Patients with AS have a higher expression of T-bet compared to healthy individuals especially in NK and CD8^+^ T cells. T-bet expressing NK cells have inflammatory functions through IFN-γ and IL-17 secretion (295). AS patients with intestinal inflammation had increased numbers of IL‐22 expressing CD3^−^CD56^+^NKp44^+^NKp46^−^ NK cells compared to other AS patients, Crohn’s disease patients as well as healthy individuals (277). NK cytotoxicity was unchanged in the synovial fluid of patients with AS compared to patients with psoriatic arthritis and juvenile chronic arthritis and in the peripheral blood of AS patients compared to healthy individuals (296). Cytotoxic activity of NK cells were also compared between HLA-B27 positive and negative AS patients and there was no significant difference between the patients groups and healthy individuals (297). NKG2A can interact with HLA-E, a non-classical MHC-class I molecule, and function as an inhibitory NK receptor, while NKG2C functions as an activating receptor. The number of CD94/NKG2A expressing NK cells were higher compared to CD94/NKG2C expressing NK cells in patients with AS. In addition, HLA-E expression was also increased in CD14^+^ monocytes of these patients, suggesting that this signaling pathway was functional in AS (298). A recent study demonstrated that increased number of CD8 expressing NK cells was associated with a better clinical response to anti-TNF-α therapies and could be suggested as a biomarker for monitorization of a better treatment response (299).

In conclusion, the genes associated with AS (HLA-B27, ERAP-1, KIRs) can potentially influence NK cell functions. There are some functional changes in the NK cell subsets indicating a NK cell response similar to Th1 pathway and certain changes in NK cell phenotype/functions may be used to predict treatment response in patients with AS.

#### Type-1 Diabetes

Type-1 diabetes is a chronic disorder of autoimmune etiology, with dramatically increased incidence during the recent years. The complete pathogenesis is still not clearly known. Pancreatic β-cells that are responsible for insulin secretion, in order to regulate blood glucose, are under immune attack. Both innate and adaptive arms of immunity contribute in disease pathogenesis, which could mainly be driven by over-activated T cells and also B cells, while studies support contribution of DCs as well as NK cells in the pathogenesis of T1D (300, 301). A number of mouse studies revealed clues about the relationship between T1D and NK cells. Increased numbers of NK cells were revealed to infiltrate the pancreatic Langerhans islets which encompass β-cells in T1D, which also had positive correlation with disease severity (302). Defects in gut NK cell numbers and also decreased cytotoxic activity were claimed to be important preceding factors for T1D onset (303). Impaired function of activating NKG2D receptor of NK cells was revealed in non-obese diabetic mouse, which was linked with down-modulation as a consequence of exposure to NKG2D ligands in pancreatic islets (304). While, infection with coxackievirus-B4 was revealed to induce an acute form of autoimmune diabetes, which could be returned by depletion of NK cells and proposed that NK-cell mediated killing of pancreatic islets *in vivo* could be initiated by coxackievirus-B4 infection (305).

In human T1D, due to toughness of having direct samples from pancreas and due to peripheral blood samples not completely reflecting the pancreatic pathogenesis, the results are conflicting. Recent onset but not long-established T1D was associated with a reduction in peripheral blood NK cell frequencies (306). When NK cytotoxic functions were investigated, some studies reported compromised cytotoxicity (307, 308) while one study reported increased NK cytotoxicity in newly diagnosed T1D patients (309). Reduced NKG2D levels were found to be associated with T1D (306). Reduced NK cell functions revealed by diminished expressions of NKp30 and NKp46 as well as reduced IFN-*γ* and perforin production capabilities were revealed in long-standing T1D patients, all of which could occur as consequences of metabolic alterations, therapy with insulin as well as exhaustion of NK cells (301, 306, 310, 311).

NK cell functions are well known to be regulated by a delicate balance of surface-expressed activating and inhibitory KIRs, which works by interacting with HLA-class 1 ligands on target cells. Contribution of KIRs in pathogenesis of autoimmune diabetes has been questioned. Accordingly, absence of 2DL2 and HLA-C1 together with absence of 2DS1 and 2DS2 were attributed as “protective” while presence of 2DL2 and HLA-C1 with the absence of 2DS1 and 2DS2 was attributed as “predisposing” factors in Latvian T1D patients (312). In another study, KIRs 2DL1, 2DS2 and 2DS4 were reported to be associated with susceptibility, while 2DS5 with protection, in Latvian patients with late-onset autoimmune diabetes (LADA). The same study reported 2DL5 and 3DL1 as predisposing while 2DS1 and 2DS3 as protective KIRs in LADA patients of Asian Indian origin, underlining both the contribution of KIRs in diabetes and importance of ethnicity in disease susceptibility (313)

In conclusion, defects in NK cell functions during viral infections could trigger autoimmunity and in turn accelerate the β-cell destruction in pancreatic Langerhans islets. KIRs and certain HLA-class I alleles could be influential in initiation and onset of diabetes, however, still, a number of questions have to be answered.

#### Psoriasis

Psoriasis is a relapsing and remitting, chronic inflammatory disease of skin, which is affecting almost 2% of the world’s population (314, 315). Psoriasis is a multifactorial disease in which genetic and environmental factors play a role in the pathogenesis but the etiopathology of the disease has not been fully clarified yet. The most accepted mechanism in the etiopathogenesis of psoriasis is the induction of inflammation with keratinocyte hyper-proliferation (316). The pathologic collaboration between innate and acquired immunity results in the production of cytokines, chemokines, and growth factors all of which cumulatively contribute in the inflammatory infiltrate seen in psoriatic plaques. T cells, DCs, macrophages and keratinocytes have roles in pathogenesis. Besides the largely documented role of T cells, emerging literature supports a potential involvement of innate immune effectors, the NK cells, in pathology (317, 318). Although the roles of NK cells against cancer or virally infected cells are well known, studies demonstrating their contribution in pathogenesis of psoriasis are still insufficient (314). Some studies indicated possible contributions of NK cells in psoriasis, with controversial results. Some studies revealed reduced CD56^dim^ and CD56^bright^ NK cell subsets in peripheral blood of patients (318), but the others indicated that there are no differences NK cell subsets between patients and healthy individuals (314). Similar situation is valid for NK cell cytotoxicity. However, studies indicated increased numbers of infiltrated NK cells into psoriatic plaques (316, 319). Several studies suggested that NK cell receptors may be associated with psoriasis (320). NKG2C is an activating receptor of NK cells that could bind to HLA-E. Wilson Liao and colleagues have identified an association between NKG2C deficiency and psoriasis (321). High NKG2C expressing NK cells can respond to virus-infected cells, and can kill autoreactive T cell, thereby could be influential in prevention from psoriasis (321). Zeng et al. revealed NKG2C deletion and HLA-E polymorphism in 611 psoriasis patients and 493 controls (320). They found that NKG2C deletion was increased, and low-expression of HLA-E*01:01 allele was associated with disease (320). Granulysin is a cytolytic antimicrobial peptide, and secreted with perforin and granzyme by NK and CD8^+^ T cells. The studies indicated increased granulysin positive cells in lesions and peripheral blood of patients and there is a link between granulysin and severe patients (315, 322). Ermis et al. showed that Granulysin rs7908 CC genotype and C allele had a protective effect against psoriasis and decreased the disease severity (322).

Some studies indicated that there is a link between resistance to disease and HLA and KIR receptor genotypes (54, 323). It was shown that above forty genes especially HLA and KIR genes have a role in pathogenesis of psoriasis (324). KIR gene family may act as a potential susceptibility factor for psoriasis (325). It was reported that activating receptors KIR2DS1 and KIR2DS2 increased the risk of developing psoriatic arthritis (PsA), particularly inhibitor receptors KIR2DL1 and KIR2DL2/3 were missing (54). Presence of KIR2DS1-HLA-C is a major risk factor for psoriasis (326). Meta-analysis studies indicated that *KIR2DL2, KIR2DS1, KIR2DS2* and *KIR2DS3* genes were positively associated with susceptibility to PsA (325), and *KIR2DS4* and *3DL1* genes appear to confer protection (327). HLA-Cw6 was found to be associated with guttate psoriasis (328, 329).

More studies are a requisite to establish a clear link between NK cells and still unclear underlying pathology of psoriasis.

## Dıscussıon and Conclusıon

**Figure 2:**
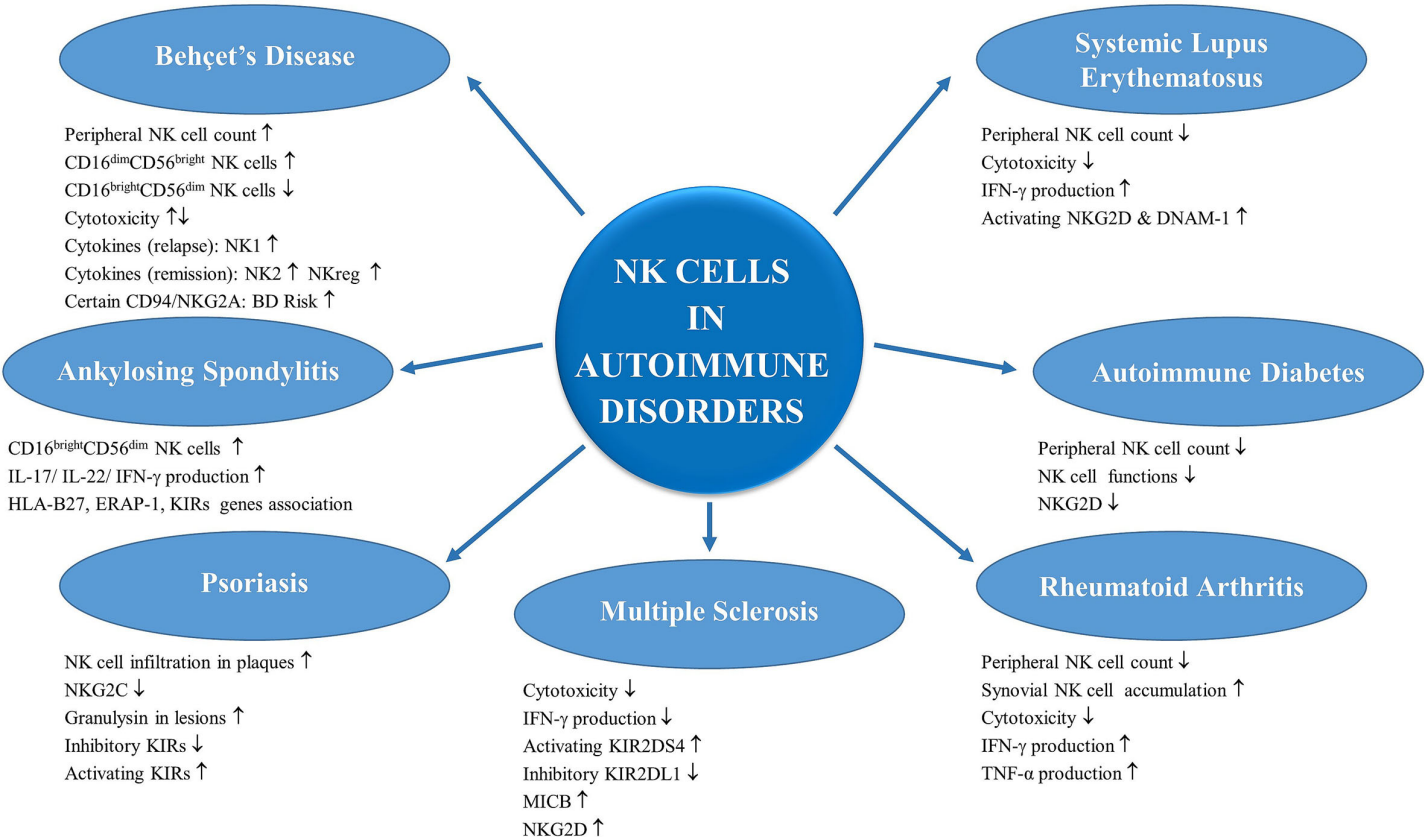
Contribution of NK cells in autoimmune disorders: NK cells have roles in underlying pathogenesis of a number of diseases with autoimmune and autoinflammatory etiology. NK cells could have both predisposing and also protective roles in these disorders thanks to their altered functional competencies and also their variable cytokine productions. The alterations in NK cell numbers and functions in Behçet’s disease, systemic lupus erythematosus, ankylosing spondylitis, autoimmune diabetes, psoriasis, rheumatoid arthritis and multiple sclerosis are summarized.

Being under focus of immunologists for decades, NK cells, their biology, functions as well as their contributions in the pathogenesis of a number of diseases are being better illuminated day by day. There is strong evidence that the innate immune system, specifically NK cells, influence subsequent adaptive immune responses. Thanks to their ability to rapidly kill abnormal cells and produce cytokines and chemokines, NK cells are positioned for a key role in regulation of autoimmune responses, and can either suppress or augment autoimmunity, directly or indirectly. NK cells could play roles with functional alterations in autoimmune conditions (**Figure 2**).

Activating and inhibitory receptors of the NK cells are essential for the regulation of NK activity and some of these NK cell receptor related genes are strongly associated with autoimmunity (330). Inhibitory receptor signals are usually generated by the binding of the MHC-class I molecules. Therefore, the functions of these receptors are especially prone to be affected in MHC-class I molecule related diseases (MHC-I-opathies) such as ankylosing spondylitis, psoriasis, and Behçet’s disease (331). Certain activating KIR receptor polymorphisms can also cause susceptibility to autoimmunity. Activating receptors KIR2DS1 and KIR2DS2 are implicated in the pathogenesis of autoimmune diseases such as in the examples of RA and psoriasis (332, 333). The expression of the ligands of the activating receptors, such as MICA also contributes to the disease pathogenesis such that alternated receptor affinty may lead to activaiton in predisposed individuals, observed in various autoimmune diseases including RA, BD and type 1 diabetes (334–336). Finally, certain KIR/HLA combinations reduce the activation threshold of the NK cells and can be protective against some infections, while the same KIR/HLA combination may also predispose autoimmunity. Individuals with HIV have a slower progression to AIDS and reduced viral loads, when they express KIR3DS1 and HLA-B Bw4-801 (337). However, the same genetic combination also causes susceptibility to autoimmunity such as in the examples of BD and AS (338, 339). The delicate balance of the activating and inhibitory KIR molecules and their ligands are important in NK cell homeostasis. Genetic and environmental factors affecting this delicate balance can potentially induce autoimmunity in susceptible individuals.

Further investigations are needed in order to unravel the roles played by NK cells, as a bridge between innate and adaptive immunity in the onset of autoimmune diseases. Expression of different subsets of NK and also ILC subsets might serve as a biomarker in the follow-up of different autoimmune diseases. The TCR-NK cells operate with a mechanism that is distinct from CAR-T cells. They can target molecules located not only on the surface of cancer cells, but inside them as well, meaning that they can reach places that are inaccessible to CAR-T cells. This technology can be adapted to target any other form of cancer and also to autoimmune diseases.

Briefly, NK cells harbor great potential both for being biomarkers and also for utilization in a number of therapeutic interventions, especially in autoimmune diseases and in cancer, all of which warrants more intense investigations to be carried out.

## Author Contributions

All authors equally contributed to this manuscript. All authors contributed to the article and approved the submitted version.

## Conflict of Interest

The authors declare that the research was conducted in the absence of any commercial or financial relationships that could be construed as a potential conflict of interest.

## References

[1] Kiessling R Klein E Pross H Wigzell H. “Natural” killer cells in the mouse. II. Cytotoxic cells with specificity for mouse Moloney leukemia cells. Characteristics of the killer cell. Eur J Immunol (1975) 5(2):117–21. 10.1002/eji.1830050209 1086218

[2] Pross HF Jondal M. Cytotoxic lymphocytes from normal donors. A functional marker of human non-T lymphocytes. Clin Exp Immunol (1975) 21(2):226–35. PMC1538269 810282

[3] Alter G Altfeld M. Perspective natural killer cells: bridging innate and adaptive immunity? IAVI Rep Newslett Int AIDS Vaccine Res (2006) 10(3):1, 6–10. 20217934

[4] Krzewski K Strominger JL. The killer’s kiss: the many functions of NK cell immunological synapses. Curr Opin Cell Biol (2008) 20(5):597–605. 10.1016/j.ceb.2008.05.006 18639449 PMC2577014

[5] Sivori S Vacca P Del Zotto G Munari E Mingari MC Moretta L. Human NK cells: surface receptors, inhibitory checkpoints, and translational applications. Cell Mol Immunol (2019) 16(5):430–41. 10.1038/s41423-019-0206-4 PMC6474200 30778167

[6] Zhang Y Huang B. The Development and Diversity of ILCs, NK Cells and Their Relevance in Health and Diseases. Adv Exp Med Biol (2017) 1024:225–44. 10.1007/978-981-10-5987-2_11 28921473

[7] Deniz G Akdis M Aktas E Blaser K Akdis CA. Human NK1 and NK2 subsets determined by purification of IFN-gamma-secreting and IFN-gamma-nonsecreting NK cells. Eur J Immunol (2002) 32(3):879–84. 10.1002/1521-4141(200203)32:3<879::AID-IMMU879>3.0.CO;2-2 11870632

[8] Deniz G Erten G Kucuksezer UC Kocacik D Karagiannidis C Aktas E. Regulatory NK cells suppress antigen-specific T cell responses. J Immunol (2008) 180(2):850–7. 10.4049/jimmunol.180.2.850 18178824

[9] Aktas E Erten G Kucuksezer UC Deniz G. Natural killer cells: versatile roles in autoimmune and infectious diseases. Expert Rev Clin Immunol (2009) 5(4):405–20. 10.1586/eci.09.27 20477037

[10] Harvey D Pointon JJ Sleator C Meenagh A Farrar C Sun JY. Analysis of killer immunoglobulin-like receptor genes in ankylosing spondylitis. Ann Rheum Diseases (2009) 68(4):595–8. 10.1136/ard.2008.095927 19019897

[11] Pallmer K Oxenius A. Recognition and Regulation of T Cells by NK Cells. Front Immunol (2016) 7:251. 10.3389/fimmu.2016.00251 27446081 PMC4919350

[12] Esen F Türkyilmaz Ö Aykut V DİReskenelİ H DenİZ G OĞUz H. Influence of İnterferon Alfa-2a Treatment on Monocyte Subsets in Patients with Uveitis. Turkish J Immunol (2020) 8(2):50–6. 10.25002/tji.2020.1261

[13] Albayrak O Oray M Can F Uludag Kirimli G Gul A Tugal-Tutkun I. Effect of Interferon alfa-2a Treatment on Adaptive and Innate Immune Systems in Patients With Behcet Disease Uveitis. Invest Ophthalmol Visual Sci (2019) 60(1):52–63. 10.1167/iovs.18-25548 30601931

[14] Mazzurana L Rao A Van Acker A Mjosberg J. The roles for innate lymphoid cells in the human immune system. Semin Immunopathol (2018) 40(4):407–19. 10.1007/s00281-018-0688-7 PMC6060849 29948108

[15] Diefenbach A Colonna M Koyasu S. Development, differentiation, and diversity of innate lymphoid cells. Immunity (2014) 41(3):354–65. 10.1016/j.immuni.2014.09.005 PMC4171710 25238093

[16] Zook EC Kee BL. Development of innate lymphoid cells. Nat Immunol (2016) 17(7):775–82. 10.1038/ni.3481 27328007

[17] Klose CS Artis D. Innate lymphoid cells as regulators of immunity, inflammation and tissue homeostasis. Nat Immunol (2016) 17(7):765–74. 10.1038/ni.3489 27328006

[18] Loza MJ Zamai L Azzoni L Rosati E Perussia B. Expression of type 1 (interferon gamma) and type 2 (interleukin-13, interleukin-5) cytokines at distinct stages of natural killer cell differentiation from progenitor cells. Blood (2002) 99(4):1273–81. 10.1182/blood.V99.4.1273 11830476

[19] Morita H Moro K Koyasu S. Innate lymphoid cells in allergic and nonallergic inflammation. J Allergy Clin Immunol (2016) 138(5):1253–64. 10.1016/j.jaci.2016.09.011 27817797

[20] Chang Y Kang SY Kim J Kang HR Kim HY. Functional Defects in Type 3 Innate Lymphoid Cells and Classical Monocytes in a Patient with Hyper-IgE Syndrome. Immune Netw (2017) 17(5):352–64. 10.4110/in.2017.17.5.352 PMC5662784 29093656

[21] Scoville SD Freud AG Caligiuri MA. Modeling Human Natural Killer Cell Development in the Era of Innate Lymphoid Cells. Front Immunol (2017) 8:360. 10.3389/fimmu.2017.00360 28396671 PMC5366880

[22] Abel AM Yang C Thakar MS Malarkannan S. Natural Killer Cells: Development, Maturation, and Clinical Utilization. Front Immunol (2018) 9:1869. 10.3389/fimmu.2018.01869 30150991 PMC6099181

[23] Freud AG Becknell B Roychowdhury S Mao HC Ferketich AK Nuovo GJ. A human CD34(+) subset resides in lymph nodes and differentiates into CD56bright natural killer cells. Immunity (2005) 22(3):295–304. 10.1016/j.immuni.2005.01.013 15780987

[24] Freud AG Yokohama A Becknell B Lee MT Mao HC Ferketich AK. Evidence for discrete stages of human natural killer cell differentiation in vivo. J Exp Med (2006) 203(4):1033–43. 10.1084/jem.20052507 PMC2118285 16606675

[25] Renoux VM Zriwil A Peitzsch C Michaelsson J Friberg D Soneji S. Identification of a Human Natural Killer Cell Lineage-Restricted Progenitor in Fetal and Adult Tissues. Immunity (2015) 43(2):394–407. 10.1016/j.immuni.2015.07.011 26287684

[26] Mace EM Hsu AP Monaco-Shawver L Makedonas G Rosen JB Dropulic L. Mutations in GATA2 cause human NK cell deficiency with specific loss of the CD56(bright) subset. Blood (2013) 121(14):2669–77. 10.1182/blood-2012-09-453969 PMC3617632 23365458

[27] Di Santo JP. Natural killer cell developmental pathways: a question of balance. Annu Rev Immunol (2006) 24:257–86. 10.1146/annurev.immunol.24.021605.090700 16551250

[28] Yu H Fehniger TA Fuchshuber P Thiel KS Vivier E Carson WE. Flt3 ligand promotes the generation of a distinct CD34(+) human natural killer cell progenitor that responds to interleukin-15. Blood (1998) 92(10):3647–57. 10.1182/blood.V92.10.3647.422k43_3647_3657 9808558

[29] Yu J Freud AG Caligiuri MA. Location and cellular stages of natural killer cell development. Trends Immunol (2013) 34(12):573–82. 10.1016/j.it.2013.07.005 PMC3852183 24055329

[30] Williams O Mok CL Norton T Harker N Kioussis D Brady HJ. Elevated Bcl-2 is not a causal event in the positive selection of T cells. Eur J Immunol (2001) 31(6):1876–82. 10.1002/1521-4141(200106)31:6<1876::AID-IMMU1876>3.0.CO;2-F 11433384

[31] Becknell B Caligiuri MA. Interleukin-2, interleukin-15, and their roles in human natural killer cells. Adv Immunol (2005) 86:209–39. 10.1016/S0065-2776(04)86006-1 15705423

[32] Montaldo E Del Zotto G Della Chiesa M Mingari MC Moretta A De Maria A. Human NK cell receptors/markers: a tool to analyze NK cell development, subsets and function. Cytometry Part A J Int Soc Anal Cytol (2013) 83(8):702–13. 10.1002/cyto.a.22302 23650273

[33] Bennett IM Zatsepina O Zamai L Azzoni L Mikheeva T Perussia B. Definition of a natural killer NKR-P1A+/CD56-/CD16- functionally immature human NK cell subset that differentiates in vitro in the presence of interleukin 12. J Exp Med (1996) 184(5):1845–56. 10.1084/jem.184.5.1845 PMC2192867 8920872

[34] Grzywacz B Kataria N Sikora M Oostendorp RA Dzierzak EA Blazar BR. Coordinated acquisition of inhibitory and activating receptors and functional properties by developing human natural killer cells. Blood (2006) 108(12):3824–33. 10.1182/blood-2006-04-020198 PMC1895469 16902150

[35] Perussia B Chen Y Loza MJ. Peripheral NK cell phenotypes: multiple changing of faces of an adapting, developing cell. Mol Immunol (2005) 42(4):385–95. 10.1016/j.molimm.2004.07.017 15607789

[36] Zamai L Del Zotto G Buccella F Gabrielli S Canonico B Artico M. Understanding the Synergy of NKp46 and Co-Activating Signals in Various NK Cell Subpopulations: Paving the Way for More Successful NK-Cell-Based Immunotherapy. Cells (2020) 9(3):753. 10.3390/cells9030753 PMC7140651 32204481

[37] Zamai L Galeotti L Del Zotto G Canonico B Mirandola P Papa S. Identification of a NCR+/NKG2D+/LFA-1(low)/CD94(-) immature human NK cell subset. Cytometry Part A J Int Soc Anal Cytol (2009) 75(11):893–901. 10.1002/cyto.a.20789 19743412

[38] Zamai L Del Zotto G Buccella F Galeotti L Canonico B Luchetti F. Cytotoxic functions and susceptibility to apoptosis of human CD56(bright) NK cells differentiated in vitro from CD34(+) hematopoietic progenitors. Cytometry Part A J Int Soc Anal Cytol (2012) 81(4):294–302. 10.1002/cyto.a.22025 22319021

[39] Freud AG Keller KA Scoville SD Mundy-Bosse BL Cheng S Youssef Y. NKp80 Defines a Critical Step during Human Natural Killer Cell Development. Cell Rep (2016) 16(2):379–91. 10.1016/j.celrep.2016.05.095 PMC4970225 27373165

[40] Vitale M Falco M Castriconi R Parolini S Zambello R Semenzato G. Identification of NKp80, a novel triggering molecule expressed by human NK cells. Eur J Immunol (2001) 31(1):233–42. 10.1002/1521-4141(200101)31:1<233::AID-IMMU233>3.0.CO;2-4 11265639

[41] Pesce S Squillario M Greppi M Loiacono F Moretta L Moretta A. New miRNA Signature Heralds Human NK Cell Subsets at Different Maturation Steps: Involvement of miR-146a-5p in the Regulation of KIR Expression. Front Immunol (2018) 9:2360. 10.3389/fimmu.2018.02360 30374356 PMC6196268

[42] Romagnani C Juelke K Falco M Morandi B D’Agostino A Costa R. CD56brightCD16- killer Ig-like receptor- NK cells display longer telomeres and acquire features of CD56dim NK cells upon activation. J Immunol (2007) 178(8):4947–55. 10.4049/jimmunol.178.8.4947 17404276

[43] Vitale M Della Chiesa M Carlomagno S Romagnani C Thiel A Moretta L. The small subset of CD56brightCD16- natural killer cells is selectively responsible for both cell proliferation and interferon-gamma production upon interaction with dendritic cells. Eur J Immunol (2004) 34(6):1715–22. 10.1002/eji.200425100 15162442

[44] Farag SS Caligiuri MA. Human natural killer cell development and biology. Blood Rev (2006) 20(3):123–37. 10.1016/j.blre.2005.10.001 16364519

[45] Poli A Michel T Theresine M Andres E Hentges F Zimmer J. CD56bright natural killer (NK) cells: an important NK cell subset. Immunology (2009) 126(4):458–65. 10.1111/j.1365-2567.2008.03027.x PMC2673358 19278419

[46] Michel T Poli A Cuapio A Briquemont B Iserentant G Ollert M. Human CD56bright NK Cells: An Update. J Immunol (2016) 196(7):2923–31. 10.4049/jimmunol.1502570 26994304

[47] Parham P Guethlein LA. Genetics of Natural Killer Cells in Human Health, Disease, and Survival. Annu Rev Immunol (2018) 36:519–48. 10.1146/annurev-immunol-042617-053149 29394121

[48] Moretta A Bottino C Vitale M Pende D Biassoni R Mingari MC. Receptors for HLA class-I molecules in human natural killer cells. Annu Rev Immunol (1996) 14:619–48. 10.1146/annurev.immunol.14.1.619 8717527

[49] Moretta A Sivori S Vitale M Pende D Morelli L Augugliaro R. Existence of both inhibitory (p58) and activatory (p50) receptors for HLA-C molecules in human natural killer cells. J Exp Med (1995) 182(3):875–84. 10.1084/jem.182.3.875 PMC2192157 7650491

[50] Biassoni R Cantoni C Falco M Verdiani S Bottino C Vitale M. The human leukocyte antigen (HLA)-C-specific “activatory” or “inhibitory” natural killer cell receptors display highly homologous extracellular domains but differ in their transmembrane and intracytoplasmic portions. J Exp Med (1996) 183(2):645–50. 10.1084/jem.183.2.645 PMC2192451 8627176

[51] Parham P. MHC class I molecules and KIRs in human history, health and survival. Nat Rev Immunol (2005) 5(3):201–14. 10.1038/nri1570 15719024

[52] Gabrielli S Ortolani C Del Zotto G Luchetti F Canonico B Buccella F. The Memories of NK Cells: Innate-Adaptive Immune Intrinsic Crosstalk. J Immunol Res (2016) 2016:1376595. 10.1155/2016/1376595 28078307 PMC5204097

[53] Hiby SE Walker JJ O’Shaughnessy KM Redman CW Carrington M Trowsdale J. Combinations of maternal KIR and fetal HLA-C genes influence the risk of preeclampsia and reproductive success. J Exp Med (2004) 200(8):957–65. 10.1084/jem.20041214 PMC2211839 15477349

[54] Nelson GW Martin MP Gladman D Wade J Trowsdale J Carrington M. Cutting edge: heterozygote advantage in autoimmune disease: hierarchy of protection/susceptibility conferred by HLA and killer Ig-like receptor combinations in psoriatic arthritis. J Immunol (2004) 173(7):4273–6. 10.4049/jimmunol.173.7.4273 15383555

[55] Vitale M Castriconi R Parolini S Pende D Hsu ML Moretta L. The leukocyte Ig-like receptor (LIR)-1 for the cytomegalovirus UL18 protein displays a broad specificity for different HLA class I alleles: analysis of LIR-1 + NK cell clones. Int Immunol (1999) 11(1):29–35. 10.1093/intimm/11.1.29 10050671

[56] Della Chiesa M Pesce S Muccio L Carlomagno S Sivori S Moretta A. Features of Memory-Like and PD-1(+) Human NK Cell Subsets. Front Immunol (2016) 7:351. 10.3389/fimmu.2016.00351 27683578 PMC5021715

[57] Moretta A Bottino C Vitale M Pende D Cantoni C Mingari MC. Activating receptors and coreceptors involved in human natural killer cell-mediated cytolysis. Annu Rev Immunol (2001) 19:197–223. 10.1146/annurev.immunol.19.1.197 11244035

[58] Sivori S Pende D Bottino C Marcenaro E Pessino A Biassoni R. NKp46 is the major triggering receptor involved in the natural cytotoxicity of fresh or cultured human NK cells. Correlation between surface density of NKp46 and natural cytotoxicity against autologous, allogeneic or xenogeneic target cells. Eur J Immunol (1999) 29(5):1656–66. 10.1002/(SICI)1521-4141(199905)29:05<1656::AID-IMMU1656>3.0.CO;2-1 10359120

[59] Vitale M Bottino C Sivori S Sanseverino L Castriconi R Marcenaro E. NKp44, a novel triggering surface molecule specifically expressed by activated natural killer cells, is involved in non-major histocompatibility complex-restricted tumor cell lysis. J Exp Med (1998) 187(12):2065–72. 10.1084/jem.187.12.2065 PMC2212362 9625766

[60] Pende D Parolini S Pessino A Sivori S Augugliaro R Morelli L. Identification and molecular characterization of NKp30, a novel triggering receptor involved in natural cytotoxicity mediated by human natural killer cells. J Exp Med (1999) 190(10):1505–16. 10.1084/jem.190.10.1505 PMC2195691 10562324

[61] Bauer S Groh V Wu J Steinle A Phillips JH Lanier LL. Pillars Article: Activation of NK Cells and T Cells by NKG2D, a Receptor for Stress-Inducible MICA. Science. 1999. 285: 727-729. J Immunol (2018) 200(7):2231–3. 10.1126/science.285.5428.727 29555676

[62] Lanier LL. NKG2D Receptor and Its Ligands in Host Defense. Cancer Immunol Res (2015) 3(6):575–82. 10.1158/2326-6066.CIR-15-0098 PMC4457299 26041808

[63] Raulet DH Gasser S Gowen BG Deng W Jung H. Regulation of ligands for the NKG2D activating receptor. Annu Rev Immunol (2013) 31:413–41. 10.1146/annurev-immunol-032712-095951 PMC4244079 23298206

[64] Sivori S Parolini S Falco M Marcenaro E Biassoni R Bottino C. 2B4 functions as a co-receptor in human NK cell activation. Eur J Immunol (2000) 30(3):787–93. 10.1002/1521-4141(200003)30:3<787::AID-IMMU787>3.0.CO;2-I 10741393

[65] Bottino C Falco M Parolini S Marcenaro E Augugliaro R Sivori S. NTB-A correction of GNTB-A], a novel SH2D1A-associated surface molecule contributing to the inability of natural killer cells to kill Epstein-Barr virus-infected B cells in X-linked lymphoproliferative disease. J Exp Med (2001) 194(3):235–46. 10.1084/jem.194.3.235 PMC2193462 11489943

[66] Shibuya A Campbell D Hannum C Yssel H Franz-Bacon K McClanahan T. DNAM-1, a novel adhesion molecule involved in the cytolytic function of T lymphocytes. Immunity (1996) 4(6):573–81. 10.1016/S1074-7613(00)70060-4 8673704

[67] Marcenaro E Augugliaro R Falco M Castriconi R Parolini S Sivori S. CD59 is physically and functionally associated with natural cytotoxicity receptors and activates human NK cell-mediated cytotoxicity. Eur J Immunol (2003) 33(12):3367–76. 10.1002/eji.200324425 14635045

[68] Sivori S Carlomagno S Moretta L Moretta A. Comparison of different CpG oligodeoxynucleotide classes for their capability to stimulate human NK cells. Eur J Immunol (2006) 36(4):961–7. 10.1002/eji.200535781 16525994

[69] Sivori S Falco M Carlomagno S Romeo E Moretta L Moretta A. Heterogeneity of TLR3 mRNA transcripts and responsiveness to poly (I:C) in human NK cells derived from different donors. Int Immunol (2007) 19(12):1341–8. 10.1093/intimm/dxm105 17962643

[70] Sivori S Falco M Carlomagno S Romeo E Soldani C Bensussan A. A novel KIR-associated function: evidence that CpG DNA uptake and shuttling to early endosomes is mediated by KIR3DL2. Blood (2010) 116(10):1637–47. 10.1182/blood-2009-12-256586 20147700

[71] Ochoa MC Minute L Rodriguez I Garasa S Perez-Ruiz E Inoges S. Antibody-dependent cell cytotoxicity: immunotherapy strategies enhancing effector NK cells. Immunol Cell Biol (2017) 95(4):347–55. 10.1038/icb.2017.6 28138156

[72] Chiossone L Dumas PY Vienne M Vivier E. Natural killer cells and other innate lymphoid cells in cancer. Nat Rev Immunol (2018) 18(11):671–88. 10.1038/s41577-018-0061-z 30209347

[73] Mittal D Vijayan D Smyth MJ. Overcoming Acquired PD-1/PD-L1 Resistance with CD38 Blockade. Cancer Discovery (2018) 8(9):1066–8. 10.1158/2159-8290.CD-18-0798 30181171

[74] Cerwenka A Lanier LL. Natural killers join the fight against cancer. Science (2018) 359(6383):1460–1. 10.1126/science.aat2184 29599226

[75] Moretta L Bottino C Pende D Castriconi R Mingari MC Moretta A. Surface NK receptors and their ligands on tumor cells. Semin Immunol (2006) 18(3):151–8. 10.1016/j.smim.2006.03.002 16730454

[76] Gauthier L Morel A Anceriz N Rossi B Blanchard-Alvarez A Grondin G. Multifunctional Natural Killer Cell Engagers Targeting NKp46 Trigger Protective Tumor Immunity. Cell (2019) 177(7):1701–13 e16. 10.1016/j.cell.2019.04.041 31155232

[77] Hrdlickova R Toloue M Tian B. RNA-Seq methods for transcriptome analysis. Wiley Interdiscip Rev RNA (2017) 8(1). 10.1002/wrna.1364 PMC5717752 27198714

[78] Kukurba KR Montgomery SB. RNA Sequencing and Analysis. Cold Spring Harbor Protoc (2015) 2015(11):951–69. 10.1101/pdb.top084970 PMC4863231 25870306

[79] Wang Z Gerstein M Snyder M. RNA-Seq: a revolutionary tool for transcriptomics. Nat Rev Genet (2009) 10(1):57–63. 10.1038/nrg2484 19015660 PMC2949280

[80] Yang C Siebert JR Burns R Zheng Y Mei A Bonacci B. Single-cell transcriptome reveals the novel role of T-bet in suppressing the immature NK gene signature. eLife (2020) 9:e51339. 10.7554/eLife.51339 32406817 PMC7255804

[81] Smith SL Kennedy PR Stacey KB Worboys JD Yarwood A Seo S. Diversity of peripheral blood human NK cells identified by single-cell RNA sequencing. Blood Advances (2020) 4(7):1388–406. 10.1182/bloodadvances.2019000699 PMC7160259 32271902

[82] Fehniger TA Wylie T Germino E Leong JW Magrini VJ Koul S. Next-generation sequencing identifies the natural killer cell microRNA transcriptome. Genome Res (2010) 20(11):1590–604. 10.1101/gr.107995.110 PMC2963822 20935160

[83] Yang C Siebert JR Burns R Gerbec ZJ Bonacci B Rymaszewski A. Heterogeneity of human bone marrow and blood natural killer cells defined by single-cell transcriptome. Nat Commun (2019) 10(1):3931. 10.1038/s41467-019-11947-7 31477722 PMC6718415

[84] Kallionpaa H Somani J Tuomela S Ullah U de Albuquerque R Lonnberg T. Early Detection of Peripheral Blood Cell Signature in Children Developing beta-Cell Autoimmunity at a Young Age. Diabetes (2019) 68(10):2024–34. 10.2337/db19-0287 31311800

[85] Arazi A Rao DA Berthier CC Davidson A Liu Y Hoover PJ. The immune cell landscape in kidneys of patients with lupus nephritis. Nat Immunol (2019) 20(7):902–14. 10.1038/s41590-019-0398-x PMC6726437 31209404

[86] Stephenson W Donlin LT Butler A Rozo C Bracken B Rashidfarrokhi A. Single-cell RNA-seq of rheumatoid arthritis synovial tissue using low-cost microfluidic instrumentation. Nat Commun (2018) 9(1):791. 10.1038/s41467-017-02659-x 29476078 PMC5824814

[87] Stabile H Fionda C Gismondi A Santoni A. Role of Distinct Natural Killer Cell Subsets in Anticancer Response. Front Immunol (2017) 8:293. 10.3389/fimmu.2017.00293 28360915 PMC5352654

[88] Smyth MJ Cretney E Kelly JM Westwood JA Street SE Yagita H. Activation of NK cell cytotoxicity. Mol Immunol (2005) 42(4):501–10. 10.1016/j.molimm.2004.07.034 15607806

[89] Fauriat C Long EO Ljunggren HG Bryceson YT. Regulation of human NK-cell cytokine and chemokine production by target cell recognition. Blood (2010) 115(11):2167–76. 10.1182/blood-2009-08-238469 PMC2844017 19965656

[90] Freeman BE Raue HP Hill AB Slifka MK. Cytokine-Mediated Activation of NK Cells during Viral Infection. J Virol (2015) 89(15):7922–31. 10.1128/JVI.00199-15 PMC4505636 25995253

[91] Khosravi-Far R Esposti MD. Death receptor signals to mitochondria. Cancer Biol Ther (2004) 3(11):1051–7. 10.4161/cbt.3.11.1173 PMC2941887 15640619

[92] Zamai L Ahmad M Bennett IM Azzoni L Alnemri ES Perussia B. Natural killer (NK) cell-mediated cytotoxicity: differential use of TRAIL and Fas ligand by immature and mature primary human NK cells. J Exp Med (1998) 188(12):2375–80. 10.1084/jem.188.12.2375 PMC2212426 9858524

[93] Nagata S Golstein P. The Fas death factor. Science (1995) 267(5203):1449–56. 10.1126/science.7533326 7533326

[94] Degli-Esposti M. To die or not to die–the quest of the TRAIL receptors. J Leukocyte Biol (1999) 65(5):535–42. 10.1002/jlb.65.5.535 10331480

[95] Guicciardi ME Gores GJ. Life and death by death receptors. FASEB J Off Publ Fed Am Societies Exp Biol (2009) 23(6):1625–37. 10.1096/fj.08-111005 PMC2698650 19141537

[96] Ashkenazi A Dixit VM. Death receptors: signaling and modulation. Science (1998) 281(5381):1305–8. 10.1126/science.281.5381.1305 9721089

[97] Crowder RN El-Deiry WS. Caspase-8 regulation of TRAIL-mediated cell death. Exp Oncol (2012) 34(3):160–4. 23070000

[98] Bao Q Shi Y. Apoptosome: a platform for the activation of initiator caspases. Cell Death Differ (2007) 14(1):56–65. 10.1038/sj.cdd.4402028 16977332

[99] Osinska I Popko K Demkow U. Perforin: an important player in immune response. Central European J Immunol (2014) 39(1):109–15. 10.5114/ceji.2014.42135 PMC4439970 26155110

[100] Gwalani LA Orange JS. Single Degranulations in NK Cells Can Mediate Target Cell Killing. J Immunol (2018) 200(9):3231–43. 10.4049/jimmunol.1701500 PMC6020067 29592963

[101] Atkinson EA Barry M Darmon AJ Shostak I Turner PC Moyer RW. Cytotoxic T lymphocyte-assisted suicide. Caspase 3 activation is primarily the result of the direct action of granzyme B. J Biol Chem (1998) 273(33):21261–6. 10.1074/jbc.273.33.21261 9694885

[102] Barry M Heibein JA Pinkoski MJ Lee SF Moyer RW Green DR. Granzyme B short-circuits the need for caspase 8 activity during granule-mediated cytotoxic T-lymphocyte killing by directly cleaving Bid. Mol Cell Biol (2000) 20(11):3781–94. 10.1128/MCB.20.11.3781-3794.2000 PMC85698 10805722

[103] Zhu Y Huang B Shi J. Fas ligand and lytic granule differentially control cytotoxic dynamics of natural killer cell against cancer target. Oncotarget (2016) 7(30):47163–72. 10.18632/oncotarget.9980 PMC5216932 27323411

[104] Juo P Kuo CJ Yuan J Blenis J. Essential requirement for caspase-8/FLICE in the initiation of the Fas-induced apoptotic cascade. Curr Biol CB (1998) 8(18):1001–8. 10.1016/S0960-9822(07)00420-4 9740801

[105] Scaffidi C Schmitz I Zha J Korsmeyer SJ Krammer PH Peter ME. Differential modulation of apoptosis sensitivity in CD95 type I and type II cells. J Biol Chem (1999) 274(32):22532–8. 10.1074/jbc.274.32.22532 10428830

[106] Green DR. Apoptotic pathways: the roads to ruin. Cell (1998) 94(6):695–8. 10.1016/S0092-8674(00)81728-6 9753316

[107] Green DR Reed JC. Mitochondria and apoptosis. Science (1998) 281(5381):1309–12. 10.1126/science.281.5381.1309 9721092

[108] Cook KD Waggoner SN Whitmire JK. NK cells and their ability to modulate T cells during virus infections. Crit Rev Immunol (2014) 34(5):359–88. 10.1615/CritRevImmunol.2014010604 PMC4266186 25404045

[109] De Maria A Bozzano F Cantoni C Moretta L. Revisiting human natural killer cell subset function revealed cytolytic CD56(dim)CD16+ NK cells as rapid producers of abundant IFN-gamma on activation. Proc Natl Acad Sci USA (2011) 108(2):728–32. 10.1073/pnas.1012356108 PMC3021076 21187373

[110] Martin-Fontecha A Thomsen LL Brett S Gerard C Lipp M Lanzavecchia A. Induced recruitment of NK cells to lymph nodes provides IFN-gamma for T(H)1 priming. Nat Immunol (2004) 5(12):1260–5. 10.1038/ni1138 15531883

[111] Deniz G Christmas SE Johnson PM. Soluble mediators and cytokines produced by human CD3- leucocyte clones from decidualized endometrium. Immunology (1996) 87(1):92–8. PMC1383973 8666442

[112] Warren HS Kinnear BF Phillips JH Lanier LL. Production of IL-5 by human NK cells and regulation of IL-5 secretion by IL-4, IL-10, and IL-12. J Immunol (1995) 154(10):5144–52. 7730620

[113] Mehrotra PT Donnelly RP Wong S Kanegane H Geremew A Mostowski HS. Production of IL-10 by human natural killer cells stimulated with IL-2 and/or IL-12. J Immunol (1998) 160(6):2637–44. 9510161

[114] van den Bosch G Preijers F Vreugdenhil A Hendriks J Maas F De Witte T. Granulocyte-macrophage colony-stimulating factor (GM-CSF) counteracts the inhibiting effect of monocytes on natural killer (NK) cells. Clin Exp Immunol (1995) 101(3):515–20. 10.1111/j.1365-2249.1995.tb03143.x PMC1553231 7664499

[115] Walzer T Dalod M Robbins SH Zitvogel L Vivier E. Natural-killer cells and dendritic cells: “l’union fait la force”. Blood (2005) 106(7):2252–8. 10.1182/blood-2005-03-1154 15933055

[116] Simonetta F Pradier A Roosnek E. T-bet and Eomesodermin in NK Cell Development, Maturation, and Function. Front Immunol (2016) 7:241. 10.3389/fimmu.2016.00241 27379101 PMC4913100

[117] Gotthardt D Sexl V. STATs in NK-Cells: The Good, the Bad, and the Ugly. Front Immunol (2016) 7:694. 10.3389/fimmu.2016.00694 28149296 PMC5241313

[118] Wang KS Ritz J Frank DA. IL-2 induces STAT4 activation in primary NK cells and NK cell lines, but not in T cells. J Immunol (1999) 162(1):299–304. 9886399

[119] Ranson T Vosshenrich CA Corcuff E Richard O Muller W Di Santo JP. IL-15 is an essential mediator of peripheral NK-cell homeostasis. Blood (2003) 101(12):4887–93. 10.1182/blood-2002-11-3392 12586624

[120] Long EO. Ready for prime time: NK cell priming by dendritic cells. Immunity (2007) 26(4):385–7. 10.1016/j.immuni.2007.04.001 17459805

[121] Degli-Esposti MA Smyth MJ. Close encounters of different kinds: dendritic cells and NK cells take centre stage. Nat Rev Immunol (2005) 5(2):112–24. 10.1038/nri1549 15688039

[122] Eduah SB Dreher E. Medicamentous modification of the urethral occlusion in instrument-determined stress incontinence in women]. Gynakologische Rundschau (1976) 16(4):257–60. 10.1159/000268631 1035875

[123] Kucuksezer UC Ozdemir C Cevhertas L Ogulur I Akdis M Akdis CA. Mechanisms of allergen-specific immunotherapy and allergen tolerance. Allergol Int Off J Japanese Soc Allergol. (2020) 69(4):549–60. 10.1016/j.alit.2020.08.002 32900655

[124] Moretta A Marcenaro E Parolini S Ferlazzo G Moretta L. NK cells at the interface between innate and adaptive immunity. Cell Death Differ (2008) 15(2):226–33. 10.1038/sj.cdd.4402170 17541426

[125] Cosan F Aktas Cetin E Akdeniz N Emrence Z Cefle A Deniz G. Natural Killer Cell Subsets and Their Functional Activity in Behcet’s Disease. Immunol Invest (2017) 46(4):419–32. 10.1080/08820139.2017.1288240 28388249

[126] Kucuksezer UC Aktas-Cetin E Bilgic-Gazioglu S Tugal-Tutkun I Gul A Deniz G. Natural killer cells dominate a Th-1 polarized response in Behcet’s disease patients with uveitis. Clin Exp Rheumatol (2015) 33(6 Suppl 94):S24–9. 25937098

[127] Tahrali I Kucuksezer UC Altintas A Uygunoglu U Akdeniz N Aktas-Cetin E. Dysfunction of CD3(-)CD16(+)CD56(dim) and CD3(-)CD16(-)CD56(bright) NK cell subsets in RR-MS patients. Clin Immunol (2018) 193:88–97. 10.1016/j.clim.2018.02.005 29448007

[128] Iyer SS Cheng G. Role of interleukin 10 transcriptional regulation in inflammation and autoimmune disease. Crit Rev Immunol (2012) 32(1):23–63. 10.1615/CritRevImmunol.v32.i1.30 22428854 PMC3410706

[129] Ferlazzo G Tsang ML Moretta L Melioli G Steinman RM Munz C. Human dendritic cells activate resting natural killer (NK) cells and are recognized via the NKp30 receptor by activated NK cells. J Exp Med (2002) 195(3):343–51. 10.1084/jem.20011149 PMC2193591 11828009

[130] Rydyznski C Daniels KA Karmele EP Brooks TR Mahl SE Moran MT. Generation of cellular immune memory and B-cell immunity is impaired by natural killer cells. Nat Commun (2015) 6:6375. 10.1038/ncomms7375 25721802 PMC4346304

[131] Schlums H Cichocki F Tesi B Theorell J Beziat V Holmes TD. Cytomegalovirus infection drives adaptive epigenetic diversification of NK cells with altered signaling and effector function. Immunity (2015) 42(3):443–56. 10.1016/j.immuni.2015.02.008 PMC4612277 25786176

[132] Sepulveda FE Maschalidi S Vosshenrich CA Garrigue A Kurowska M Menasche G. A novel immunoregulatory role for NK-cell cytotoxicity in protection from HLH-like immunopathology in mice. Blood (2015) 125(9):1427–34. 10.1182/blood-2014-09-602946 25525117

[133] Kirino Y Bertsias G Ishigatsubo Y Mizuki N Tugal-Tutkun I Seyahi E. Genome-wide association analysis identifies new susceptibility loci for Behcet’s disease and epistasis between HLA-B*51 and ERAP1. Nat Genet (2013) 45(2):202–7. 10.1038/ng.2520 PMC3810947 23291587

[134] Kirino Y Zhou Q Ishigatsubo Y Mizuki N Tugal-Tutkun I Seyahi E. Targeted resequencing implicates the familial Mediterranean fever gene MEFV and the toll-like receptor 4 gene TLR4 in Behcet disease. Proc Natl Acad Sci USA (2013) 110(20):8134–9. 10.1073/pnas.1306352110 PMC3657824 23633568

[135] Remmers EF Cosan F Kirino Y Ombrello MJ Abaci N Satorius C. Genome-wide association study identifies variants in the MHC class I, IL10, and IL23R-IL12RB2 regions associated with Behcet’s disease. Nat Genet (2010) 42(8):698–702. 10.1038/ng.625 20622878 PMC2923807

[136] Mizuki N Meguro A Ota M Ohno S Shiota T Kawagoe T. Genome-wide association studies identify IL23R-IL12RB2 and IL10 as Behcet’s disease susceptibility loci. Nat Genet (2010) 42(8):703–6. 10.1038/ng.624 20622879

[137] Ombrello MJ Kirino Y de Bakker PI Gul A Kastner DL Remmers EF. Behcet disease-associated MHC class I residues implicate antigen binding and regulation of cell-mediated cytotoxicity. Proc Natl Acad Sci USA (2014) 111(24):8867–72. 10.1073/pnas.1406575111 PMC4066484 24821759

[138] Wallace GR Kondeatis E Vaughan RW Verity DH Chen Y Fortune F. IL-10 genotype analysis in patients with Behcet’s disease. Hum Immunol (2007) 68(2):122–7. 10.1016/j.humimm.2006.11.010 17321902

[139] Gul A. Pathogenesis of Behcet’s disease: autoinflammatory features and beyond. Semin Immunopathol (2015) 37(4):413–8. 10.1007/s00281-015-0502-8 26068404

[140] Kaneko F Takahashi Y Muramatsu R Adachi K Miura Y Nakane A. Natural killer cell numbers and function in peripheral lymphoid cells in Behcet’s disease. Br J Dermatol (1985) 113(3):313–8. 10.1111/j.1365-2133.1985.tb02083.x 4063167

[141] Suzuki Y Hoshi K Matsuda T Mizushima Y. Increased peripheral blood gamma delta+ T cells and natural killer cells in Behcet’s disease. J Rheumatol (1992) 19(4):588–92. 1534375

[142] Aktas Cetin E Cosan F Kucuksezer UC Bilgic S Cagatay Y Gul A. Behcet’s disease: immunological relevance with arthritis of ankylosing spondylitis. Rheumatol Int (2013) 33(3):733–41. 10.1007/s00296-012-2446-9 22576660

[143] Sakly K Lahmar R Nefzi F Hammami S Harzallah O Sakly N. Phenotypic abnormalities of peripheral blood mononuclear cells in patients with Behcet’s disease and association with HLA-B51 expression. Immunol Invest (2014) 43(5):463–78. 10.3109/08820139.2014.886260 24661088

[144] Hasan MS Ryan PL Bergmeier LA Fortune F. Circulating NK cells and their subsets in Behcet’s disease. Clin Exp Immunol (2017) 188(2):311–22. 10.1111/cei.12939 PMC5383445 28170096

[145] Dittmer J Bennett M. Marrow-thymus cell radiation chimeras accept allogeneic rat hearts. Transplant Proc (1977) 9(1):895–900. 325820

[146] Onder M Bozkurt M Gurer MA Gulekon A Sezgin P Imir T. Natural cellular cytotoxicity in Behcet’s disease. J Dermatol (1994) 21(4):239–43. 10.1111/j.1346-8138.1994.tb01729.x 8056895

[147] Hamzaoui K Berraies A Kaabachi W Ammar J Hamzaoui A. Pulmonary manifestations in Behcet disease: impaired natural killer cells activity. Multidiscip Respir Med (2013) 8(1):29. 10.1186/2049-6958-8-29 23556512 PMC3637264

[148] Bonacini M Soriano A Zerbini A Calo E Cimino L Muratore F. Higher Frequencies of Lymphocytes Expressing the Natural Killer Group 2D Receptor in Patients With Behcet Disease. Front Immunol (2018) 9:2157. 10.3389/fimmu.2018.02157 30319620 PMC6167483

[149] Aktas Cetin E Cosan F Cefle A Deniz G. IL-22-secreting Th22 and IFN-gamma-secreting Th17 cells in Behcet’s disease. Modern Rheumatol (2014) 24(5):802–7. 10.3109/14397595.2013.879414 24498963

[150] Ben Ahmed M Houman H Miled M Dellagi K Louzir H. Involvement of chemokines and Th1 cytokines in the pathogenesis of mucocutaneous lesions of Behcet’s disease. Arthritis Rheum (2004) 50(7):2291–5. 10.1002/art.20334 15248229

[151] Frassanito MA Dammacco R Cafforio P Dammacco F. Th1 polarization of the immune response in Behcet’s disease: a putative pathogenetic role of interleukin-12. Arthritis Rheum (1999) 42(9):1967–74. 10.1002/1529-0131(199909)42:9<1967::AID-ANR24>3.0.CO;2-Z 10513814

[152] Okamura H Kashiwamura S Tsutsui H Yoshimoto T Nakanishi K. Regulation of interferon-gamma production by IL-12 and IL-18. Curr Opin Immunol (1998) 10(3):259–64. 10.1016/S0952-7915(98)80163-5 9638361

[153] Yamaguchi Y Takahashi H Satoh T Okazaki Y Mizuki N Takahashi K. Natural killer cells control a T-helper 1 response in patients with Behcet’s disease. Arthritis Res Ther (2010) 12(3):R80. 10.1186/ar3005 20459787 PMC2911862

[154] Sanjanwala B Draghi M Norman PJ Guethlein LA Parham P. Polymorphic sites away from the Bw4 epitope that affect interaction of Bw4+ HLA-B with KIR3DL1. J Immunol (2008) 181(9):6293–300. 10.4049/jimmunol.181.9.6293 PMC2634290 18941220

[155] Petrushkin H Hasan MS Stanford MR Fortune F Wallace GR. Behcet’s Disease: Do Natural Killer Cells Play a Significant Role? Front Immunol (2015) 6:134. 10.3389/fimmu.2015.00134 25852697 PMC4371743

[156] Erer B Takeuchi M Ustek D Tugal-Tutkun I Seyahi E Ozyazgan Y. Evaluation of KIR3DL1/KIR3DS1 polymorphism in Behcet’s disease. Genes Immunity (2016) 17(7):396–9. 10.1038/gene.2016.36 PMC5590678 27708262

[157] Takeno M Shimoyama Y Kashiwakura J Nagafuchi H Sakane T Suzuki N. Abnormal killer inhibitory receptor expression on natural killer cells in patients with Behcet’s disease. Rheumatol Int (2004) 24(4):212–6. 10.1007/s00296-003-0352-x 12879269

[158] Karasneh J Gul A Ollier WE Silman AJ Worthington J. Whole-genome screening for susceptibility genes in multicase families with Behcet’s disease. Arthritis Rheum (2005) 52(6):1836–42. 10.1002/art.21060 15934084

[159] Sobanov Y Glienke J Brostjan C Lehrach H Francis F Hofer E. Linkage of the NKG2 and CD94 receptor genes to D12S77 in the human natural killer gene complex. Immunogenetics (1999) 49(2):99–105. 10.1007/s002510050468 9887346

[160] Seo J Park JS Nam JH Bang D Sohn S Lee ES. Association of CD94/NKG2A, CD94/NKG2C, and its ligand HLA-E polymorphisms with Behcet’s disease. Tissue Antigens (2007) 70(4):307–13. 10.1111/j.1399-0039.2007.00907.x 17767552

[161] Yang Y Tan H Deng B Yu H Su G Hu J. Genetic polymorphisms of C-type lectin receptors in Behcet’s disease in a Chinese Han population. Sci Rep (2017) 7(1):5348. 10.1038/s41598-017-05877-x 28706259 PMC5509750

[162] Saruhan-Direskeneli G Uyar FA Cefle A Onder SC Eksioglu-Demiralp E Kamali S. Expression of KIR and C-type lectin receptors in Behcet’s disease. Rheumatol (Oxford) (2004) 43(4):423–7. 10.1093/rheumatology/keh063 14679294

[163] Yazici H Fresko I Yurdakul S. Behcet’s syndrome: disease manifestations, management, and advances in treatment. Nat Clin Pract Rheumatol (2007) 3(3):148–55. 10.1038/ncprheum0436 17334337

[164] Baecher-Allan C Kaskow BJ Weiner HL. Multiple Sclerosis: Mechanisms and Immunotherapy. Neuron (2018) 97(4):742–68. 10.1016/j.neuron.2018.01.021 29470968

[165] Lublin FD Reingold SC. Defining the clinical course of multiple sclerosis: results of an international survey. National Multiple Sclerosis Society (USA) Advisory Committee on Clinical Trials of New Agents in Multiple Sclerosis. Neurology (1996) 46(4):907–11. 10.1212/WNL.46.4.907 8780061

[166] Dendrou CA Fugger L Friese MA. Immunopathology of multiple sclerosis. Nat Rev Immunol (2015) 15(9):545–58. 10.1038/nri3871 26250739

[167] Steinman L. Multiple sclerosis: a two-stage disease. Nat Immunol (2001) 2(9):762–4. 10.1038/ni0901-762 11526378

[168] Aktas O Ullrich O Infante-Duarte C Nitsch R Zipp F. Neuronal damage in brain inflammation. Arch Neurol (2007) 64(2):185–9. 10.1001/archneur.64.2.185 17296833

[169] Park H Li Z Yang XO Chang SH Nurieva R Wang YH. A distinct lineage of CD4 T cells regulates tissue inflammation by producing interleukin 17. Nat Immunol (2005) 6(11):1133–41. 10.1038/ni1261 PMC1618871 16200068

[170] Salou M Nicol B Garcia A Laplaud DA. Involvement of CD8(+) T Cells in Multiple Sclerosis. Front Immunol (2015) 6:604. 10.3389/fimmu.2015.00604 26635816 PMC4659893

[171] Machado-Santos J Saji E Troscher AR Paunovic M Liblau R Gabriely G. The compartmentalized inflammatory response in the multiple sclerosis brain is composed of tissue-resident CD8+ T lymphocytes and B cells. Brain J Neurol (2018) 141(7):2066–82. 10.1093/brain/awy151 PMC6022681 29873694

[172] Gold R Giovannoni G Selmaj K Havrdova E Montalban X Radue EW. Daclizumab high-yield process in relapsing-remitting multiple sclerosis (SELECT): a randomised, double-blind, placebo-controlled trial. Lancet (2013) 381(9884):2167–75. 10.1016/S0140-6736(12)62190-4 23562009

[173] Giovannoni G Gold R Selmaj K Havrdova E Montalban X Radue EW. Daclizumab high-yield process in relapsing-remitting multiple sclerosis (SELECTION): a multicentre, randomised, double-blind extension trial. Lancet Neurol (2014) 13(5):472–81. 10.1016/S1474-4422(14)70039-0 24656609

[174] Lunemann JD Munz C. Do natural killer cells accelerate or prevent autoimmunity in multiple sclerosis? Brain J Neurol (2008) 131(Pt 7):1681–3. 10.1093/brain/awn132 18586760

[175] Gandhi R Laroni A Weiner HL. Role of the innate immune system in the pathogenesis of multiple sclerosis. J Neuroimmunol (2010) 221(1-2):7–14. 10.1016/j.jneuroim.2009.10.015 19931190 PMC2854189

[176] Trachtenberg EA. Understanding the role of natural killer cell receptors and their human leukocyte antigen ligands in multiple sclerosis. Ann Neurol (2009) 65(6):626–8. 10.1002/ana.21747 19557875

[177] Lorentzen AR Karlsen TH Olsson M Smestad C Mero IL Woldseth B. Killer immunoglobulin-like receptor ligand HLA-Bw4 protects against multiple sclerosis. Ann Neurol (2009) 65(6):658–66. 10.1002/ana.21695 19630074

[178] Rizzo R Gentili V Casetta I Caselli E De Gennaro R Granieri E. Altered natural killer cells’ response to herpes virus infection in multiple sclerosis involves KIR2DL2 expression. J Neuroimmunol (2012) 251(1-2):55–64. 10.1016/j.jneuroim.2012.07.004 22871633

[179] Rizzo R Bortolotti D Fainardi E Gentili V Bolzani S Baldi E. KIR2DL2 inhibitory pathway enhances Th17 cytokine secretion by NK cells in response to herpesvirus infection in multiple sclerosis patients. J Neuroimmunol (2016) 294:1–5. 10.1016/j.jneuroim.2016.03.007 27138091

[180] Kaur G Trowsdale J Fugger L. Natural killer cells and their receptors in multiple sclerosis. Brain J Neurol (2013) 136(Pt 9):2657–76. 10.1093/brain/aws159 PMC3754456 22734127

[181] Eagle RA Trowsdale J. Promiscuity and the single receptor: NKG2D. Nat Rev Immunol (2007) 7(9):737–44. 10.1038/nri2144 17673918

[182] Zafirova B Wensveen FM Gulin M Polic B. Regulation of immune cell function and differentiation by the NKG2D receptor. Cell Mol Life Sci CMLS (2011) 68(21):3519–29. 10.1007/s00018-011-0797-0 PMC3192283 21898152

[183] Fernandez-Morera JL Rodriguez-Rodero S Lahoz C Tunon A Astudillo A Garcia-Suarez O. Soluble MHC class I chain-related protein B serum levels correlate with disease activity in relapsing-remitting multiple sclerosis. Hum Immunol (2008) 69(4-5):235–40. 10.1016/j.humimm.2008.01.021 18486757

[184] Fernandez-Morera JL Rodriguez-Rodero S Tunon A Martinez-Borra J Vidal-Castineira JR Lopez-Vazquez A. Genetic influence of the nonclassical major histocompatibility complex class I molecule MICB in multiple sclerosis susceptibility. Tissue Antigens (2008) 72(1):54–9. 10.1111/j.1399-0039.2008.01066.x 18588574

[185] Cerboni C Zingoni A Cippitelli M Piccoli M Frati L Santoni A. Antigen-activated human T lymphocytes express cell-surface NKG2D ligands via an ATM/ATR-dependent mechanism and become susceptible to autologous NK- cell lysis. Blood (2007) 110(2):606–15. 10.1182/blood-2006-10-052720 17405908

[186] Nielsen N Odum N Urso B Lanier LL Spee P. Cytotoxicity of CD56(bright) NK cells towards autologous activated CD4+ T cells is mediated through NKG2D, LFA-1 and TRAIL and dampened via CD94/NKG2A. PloS One (2012) 7(2):e31959. 10.1371/journal.pone.0031959 22384114 PMC3284517

[187] Groh V Wu J Yee C Spies T. Tumour-derived soluble MIC ligands impair expression of NKG2D and T-cell activation. Nature (2002) 419(6908):734–8. 10.1038/nature01112 12384702

[188] Molfetta R Quatrini L Santoni A Paolini R. Regulation of NKG2D-Dependent NK Cell Functions: The Yin and the Yang of Receptor Endocytosis. Int J Mol Sci (2017) 18(8):1677. 10.3390/ijms18081677 PMC5578067 28767057

[189] Tahrali I Kucuksezer UC Akdeniz N Altintas A Uygunoglu U Aktas-Cetin E. CD3(-)CD56(+) NK cells display an inflammatory profile in RR-MS patients. Immunol Lett (2019) 216:63–9. 10.1016/j.imlet.2019.10.006 31589897

[190] Jiang W Chai NR Maric D Bielekova B. Unexpected role for granzyme K in CD56bright NK cell-mediated immunoregulation of multiple sclerosis. J Immunol (2011) 187(2):781–90. 10.4049/jimmunol.1100789 PMC3131478 21666061

[191] Chanvillard C Jacolik RF Infante-Duarte C Nayak RC. The role of natural killer cells in multiple sclerosis and their therapeutic implications. Front Immunol (2013) 4:63. 10.3389/fimmu.2013.00063 23493880 PMC3595639

[192] Morse RH Seguin R McCrea EL Antel JP. NK cell-mediated lysis of autologous human oligodendrocytes. J Neuroimmunol (2001) 116(1):107–15. 10.1016/S0165-5728(01)00289-2 11311336

[193] Saikali P Antel JP Newcombe J Chen Z Freedman M Blain M. NKG2D-mediated cytotoxicity toward oligodendrocytes suggests a mechanism for tissue injury in multiple sclerosis. J Neurosci Off J Soc Neurosci (2007) 27(5):1220–8. 10.1523/JNEUROSCI.4402-06.2007 PMC6673175 17267578

[194] Benczur M Petranyl GG Palffy G Varga M Talas M Kotsy B. Dysfunction of natural killer cells in multiple sclerosis: a possible pathogenetic factor. Clin Exp Immunol (1980) 39(3):657–62. PMC1538133 6155232

[195] Kastrukoff LF Lau A Wee R Zecchini D White R Paty DW. Clinical relapses of multiple sclerosis are associated with ‘novel’ valleys in natural killer cell functional activity. J Neuroimmunol (2003) 145(1-2):103–14. 10.1016/j.jneuroim.2003.10.001 14644036

[196] Gross CC Schulte-Mecklenbeck A Runzi A Kuhlmann T Posevitz-Fejfar A Schwab N. Impaired NK-mediated regulation of T-cell activity in multiple sclerosis is reconstituted by IL-2 receptor modulation. Proc Natl Acad Sci USA (2016) 113(21):E2973–82. 10.1073/pnas.1524924113 PMC4889377 27162345

[197] Kastrukoff LF Morgan NG Zecchini D White R Petkau AJ Satoh J. A role for natural killer cells in the immunopathogenesis of multiple sclerosis. J Neuroimmunol (1998) 86(2):123–33. 10.1016/s0165-5728(98)00014-9 9663557

[198] Xu W Fazekas G Hara H Tabira T. Mechanism of natural killer (NK) cell regulatory role in experimental autoimmune encephalomyelitis. J Neuroimmunol (2005) 163(1-2):24–30. 10.1016/j.jneuroim.2005.02.011 15885305

[199] Zhang B Yamamura T Kondo T Fujiwara M Tabira T. Regulation of experimental autoimmune encephalomyelitis by natural killer (NK) cells. J Exp Med (1997) 186(10):1677–87. 10.1084/jem.186.10.1677 PMC2199138 9362528

[200] Huang D Shi FD Jung S Pien GC Wang J Salazar-Mather TP. The neuronal chemokine CX3CL1/fractalkine selectively recruits NK cells that modify experimental autoimmune encephalomyelitis within the central nervous system. FASEB J Off Publ Fed Am Societies Exp Biol (2006) 20(7):896–905. 10.1096/fj.05-5465com 16675847

[201] Fierz W. Multiple sclerosis: an example of pathogenic viral interaction? Virol J (2017) 14(1):42. 10.1186/s12985-017-0719-3 28241767 PMC5330019

[202] Virtanen JO Jacobson S. Viruses and multiple sclerosis. CNS Neurol Disord Drug Targets (2012) 11(5):528–44. 10.2174/187152712801661220 PMC4758194 22583435

[203] Madsen C. The innovative development in interferon beta treatments of relapsing-remitting multiple sclerosis. Brain Behav (2017) 7(6):e00696. 10.1002/brb3.696 28638705 PMC5474703

[204] Tarlinton RE Martynova E Rizvanov AA Khaiboullina S Verma S. Role of Viruses in the Pathogenesis of Multiple Sclerosis. Viruses (2020) 12(6):643. 10.3390/v12060643 PMC7354629 32545816

[205] Cooper MA Fehniger TA Turner SC Chen KS Ghaheri BA Ghayur T. Human natural killer cells: a unique innate immunoregulatory role for the CD56(bright) subset. Blood (2001) 97(10):3146–51. 10.1182/blood.V97.10.3146 11342442

[206] Morandi F Horenstein AL Chillemi A Quarona V Chiesa S Imperatori A. CD56brightCD16- NK Cells Produce Adenosine through a CD38-Mediated Pathway and Act as Regulatory Cells Inhibiting Autologous CD4+ T Cell Proliferation. J Immunol (2015) 195(3):965–72. 10.4049/jimmunol.1500591 26091716

[207] Laroni A Gandhi R Beynon V Weiner HL. IL-27 imparts immunoregulatory function to human NK cell subsets. PloS One (2011) 6(10):e26173. 10.1371/journal.pone.0026173 22039443 PMC3198386

[208] Laroni A Uccelli A. CD56bright Natural Killer Cells: A Possible Biomarker of Different Treatments in Multiple Sclerosis. J Clin Med (2020) 9(5):1450. 10.3390/jcm9051450 PMC7291063 32414131

[209] Fogel LA Yokoyama WM French AR. Natural killer cells in human autoimmune disorders. Arthritis Res Ther (2013) 15(4):216. 10.1186/ar4232 23856014 PMC3979027

[210] Bielekova B Catalfamo M Reichert-Scrivner S Packer A Cerna M Waldmann TA. Regulatory CD56(bright) natural killer cells mediate immunomodulatory effects of IL-2Ralpha-targeted therapy (daclizumab) in multiple sclerosis. Proc Natl Acad Sci USA (2006) 103(15):5941–6. 10.1073/pnas.0601335103 PMC1458677 16585503

[211] Bielekova B Richert N Herman ML Ohayon J Waldmann TA McFarland H. Intrathecal effects of daclizumab treatment of multiple sclerosis. Neurology (2011) 77(21):1877–86. 10.1212/WNL.0b013e318239f7ef PMC3246406 22076546

[212] Martinez-Rodriguez JE Lopez-Botet M Munteis E Rio J Roquer J Montalban X. Natural killer cell phenotype and clinical response to interferon-beta therapy in multiple sclerosis. Clin Immunol (2011) 141(3):348–56. 10.1016/j.clim.2011.09.006 21992960

[213] Gross CC Ahmetspahic D Ruck T Schulte-Mecklenbeck A Schwarte K Jorgens S. Alemtuzumab treatment alters circulating innate immune cells in multiple sclerosis. Neurol (R) Neuroimmunol Neuroinflamm (2016) 3(6):e289. 10.1212/NXI.0000000000000289 PMC5063395 27766281

[214] Saraste M Irjala H Airas L. Expansion of CD56Bright natural killer cells in the peripheral blood of multiple sclerosis patients treated with interferon-beta. Neurol Sci Off J Ital Neurol Soc Ital Soc Clin Neurophysiol (2007) 28(3):121–6. 10.1007/s10072-007-0803-3 17603762

[215] Kuwabara T Ishikawa F Kondo M Kakiuchi T. The Role of IL-17 and Related Cytokines in Inflammatory Autoimmune Diseases. Mediators Inflamm (2017) 2017:3908061. 10.1155/2017/3908061 28316374 PMC5337858

[216] Olsson T. Cytokines in neuroinflammatory disease: role of myelin autoreactive T cell production of interferon-gamma. J Neuroimmunol (1992) 40(2-3):211–8. 10.1016/0165-5728(92)90135-8 1430152

[217] Lunemann A Tackenberg B DeAngelis T da Silva RB Messmer B Vanoaica LD. Impaired IFN-gamma production and proliferation of NK cells in multiple sclerosis. Int Immunol (2011) 23(2):139–48. 10.1093/intimm/dxq463 PMC3030728 21212154

[218] Tsokos GC. Systemic lupus erythematosus. New Engl J Med (2011) 365(22):2110–21. 10.1056/NEJMra1100359 22129255

[219] Zhang CX Wang HY Yin L Mao YY Zhou W. Immunometabolism in the pathogenesis of systemic lupus erythematosus. J Trans Autoimmunity (2020) 3:100046. 10.1016/j.jtauto.2020.100046 PMC7388408 32743527

[220] Justiz Vaillant AA Goyal A Bansal P Varacallo M. Systemic Lupus Erythematosus (SLE). Treasure Island (FL: StatPearls (2020). 30571026

[221] Shao WH Cohen PL. Disturbances of apoptotic cell clearance in systemic lupus erythematosus. Arthritis Res Ther (2011) 13(1):202. 10.1186/ar3206 21371352 PMC3157636

[222] Mistry P Kaplan MJ. Cell death in the pathogenesis of systemic lupus erythematosus and lupus nephritis. Clin Immunol (2017) 185:59–73. 10.1016/j.clim.2016.08.010 27519955 PMC5299061

[223] Mackern-Oberti JP Llanos C Riedel CA Bueno SM Kalergis AM. Contribution of dendritic cells to the autoimmune pathology of systemic lupus erythematosus. Immunology (2015) 146(4):497–507. 10.1111/imm.12504 26173489 PMC4693898

[224] Guiducci C Gong M Xu Z Gill M Chaussabel D Meeker T. TLR recognition of self nucleic acids hampers glucocorticoid activity in lupus. Nature (2010) 465(7300):937–41. 10.1038/nature09102 PMC2964153 20559388

[225] Isenberg DA Manson JJ Ehrenstein MR Rahman A. Fifty years of anti-ds DNA antibodies: are we approaching journey’s end? Rheumatol (Oxford) (2007) 46(7):1052–6. 10.1093/rheumatology/kem112 17500073

[226] Aringer M. Inflammatory markers in systemic lupus erythematosus. J Autoimmunity (2020) 110:102374. 10.1016/j.jaut.2019.102374 31812331

[227] Sang A Zheng YY Morel L. Contributions of B cells to lupus pathogenesis. Mol Immunol (2014) 62(2):329–38. 10.1016/j.molimm.2013.11.013 PMC4053496 24332482

[228] Clynes R Dumitru C Ravetch JV. Uncoupling of immune complex formation and kidney damage in autoimmune glomerulonephritis. Science (1998) 279(5353):1052–4. 10.1126/science.279.5353.1052 9461440

[229] Herrada AA Escobedo N Iruretagoyena M Valenzuela RA Burgos PI Cuitino L. Innate Immune Cells’ Contribution to Systemic Lupus Erythematosus. Front Immunol (2019) 10:772. 10.3389/fimmu.2019.00772 31037070 PMC6476281

[230] Park YW Kee SJ Cho YN Lee EH Lee HY Kim EM. Impaired differentiation and cytotoxicity of natural killer cells in systemic lupus erythematosus. Arthritis Rheum (2009) 60(6):1753–63. 10.1002/art.24556 19479851

[231] Riccieri V Spadaro A Parisi G Taccari E Moretti T Bernardini G. Down-regulation of natural killer cells and of gamma/delta T cells in systemic lupus erythematosus. Does it correlate to autoimmunity and to laboratory indices of disease activity? Lupus (2000) 9(5):333–7. 10.1191/096120300678828460 10878724

[232] Erkeller-Yuksel FM Lydyard PM Isenberg DA. Lack of NK cells in lupus patients with renal involvement. Lupus (1997) 6(9):708–12. 10.1177/096120339700600905 9412985

[233] Huang Z Fu B Zheng SG Li X Sun R Tian Z. Involvement of CD226+ NK cells in immunopathogenesis of systemic lupus erythematosus. J Immunol (2011) 186(6):3421–31. 10.4049/jimmunol.1000569 PMC3097030 21296979

[234] Fitzgerald-Bocarsly P Dai J Singh S. Plasmacytoid dendritic cells and type I IFN: 50 years of convergent history. Cytokine Growth factor Rev (2008) 19(1):3–19. 10.1016/j.cytogfr.2007.10.006 18248767 PMC2277216

[235] Hagberg N Berggren O Leonard D Weber G Bryceson YT Alm GV. IFN-alpha production by plasmacytoid dendritic cells stimulated with RNA-containing immune complexes is promoted by NK cells via MIP-1beta and LFA-1. J Immunol (2011) 186(9):5085–94. 10.4049/jimmunol.1003349 21430220

[236] Eloranta ML Lovgren T Finke D Mathsson L Ronnelid J Kastner B. Regulation of the interferon-alpha production induced by RNA-containing immune complexes in plasmacytoid dendritic cells. Arthritis Rheum (2009) 60(8):2418–27. 10.1002/art.24686 19644885

[237] Green MR Kennell AS Larche MJ Seifert MH Isenberg DA Salaman MR. Natural killer cell activity in families of patients with systemic lupus erythematosus: demonstration of a killing defect in patients. Clin Exp Immunol (2005) 141(1):165–73. 10.1111/j.1365-2249.2005.02822.x PMC1809425 15958083

[238] Yabuhara A Yang FC Nakazawa T Iwasaki Y Mori T Koike K. A killing defect of natural killer cells as an underlying immunologic abnormality in childhood systemic lupus erythematosus. J Rheumatol (1996) 23(1):171–7. 8838528

[239] Hervier B Beziat V Haroche J Mathian A Lebon P Ghillani-Dalbin P. Phenotype and function of natural killer cells in systemic lupus erythematosus: excess interferon-gamma production in patients with active disease. Arthritis Rheum (2011) 63(6):1698–706. 10.1002/art.30313 21370226

[240] Alter G Malenfant JM Altfeld M. CD107a as a functional marker for the identification of natural killer cell activity. J Immunol Methods (2004) 294(1-2):15–22. 10.1016/j.jim.2004.08.008 15604012

[241] Ye Z Ma N Zhao L Jiang ZY Jiang YF. Differential expression of natural killer activating and inhibitory receptors in patients with newly diagnosed systemic lupus erythematosus. Int J Rheum Diseases (2016) 19(6):613–21. 10.1111/1756-185X.12289 24617980

[242] Hodge DL Berthet C Coppola V Kastenmuller W Buschman MD Schaughency PM. IFN-gamma AU-rich element removal promotes chronic IFN-gamma expression and autoimmunity in mice. J Autoimmunity (2014) 53:33–45. 10.1016/j.jaut.2014.02.003 24583068 PMC4148478

[243] Liu M Liu J Hao S Wu P Zhang X Xiao Y. Higher activation of the interferon-gamma signaling pathway in systemic lupus erythematosus patients with a high type I IFN score: relation to disease activity. Clin Rheumatol (2018) 37(10):2675–84. 10.1007/s10067-018-4138-7 29774490

[244] Huang X Li J Dorta-Estremera S Di Domizio J Anthony SM Watowich SS. Neutrophils Regulate Humoral Autoimmunity by Restricting Interferon-gamma Production via the Generation of Reactive Oxygen Species. Cell Rep (2015) 12(7):1120–32. 10.1016/j.celrep.2015.07.021 PMC4545388 26257170

[245] Hagberg N Theorell J Hjorton K Spee P Eloranta ML Bryceson YT. Functional anti-CD94/NKG2A and anti-CD94/NKG2C autoantibodies in patients with systemic lupus erythematosus. Arthritis Rheumatol (2015) 67(4):1000–11. 10.1002/art.38999 25510434

[246] Segerberg F Lundtoft C Reid S Hjorton K Leonard D Nordmark G. Autoantibodies to Killer Cell Immunoglobulin-Like Receptors in Patients With Systemic Lupus Erythematosus Induce Natural Killer Cell Hyporesponsiveness. Front Immunol (2019) 10:2164. 10.3389/fimmu.2019.02164 31572377 PMC6749077

[247] Puxeddu I Bongiorni F Chimenti D Bombardieri S Moretta A Bottino C. Cell surface expression of activating receptors and co-receptors on peripheral blood NK cells in systemic autoimmune diseases. Scand J Rheumatol (2012) 41(4):298–304. 10.3109/03009742.2011.648657 22632143

[248] Li WX Pan HF Hu JL Wang CZ Zhang N Li J. Assay of T- and NK-cell subsets and the expression of NKG2A and NKG2D in patients with new-onset systemic lupus erythematosus. Clin Rheumatol (2010) 29(3):315–23. 10.1007/s10067-009-1322-9 20012119

[249] Schepis D Gunnarsson I Eloranta ML Lampa J Jacobson SH Karre K. Increased proportion of CD56bright natural killer cells in active and inactive systemic lupus erythematosus. Immunology (2009) 126(1):140–6. 10.1111/j.1365-2567.2008.02887.x PMC2632704 18564343

[250] Spada R Rojas JM Barber DF. Recent findings on the role of natural killer cells in the pathogenesis of systemic lupus erythematosus. J Leukocyte Biol (2015) 98(4):479–87. 10.1189/jlb.4RU0315-081RR 26216938

[251] Liu M Liu J Zhang X Xiao Y Jiang G Huang X. Activation status of CD56(dim) natural killer cells is associated with disease activity of patients with systemic lupus erythematosus. Clin Rheumatol (2020). 10.1007/s10067-020-05306-x 32797360

[252] Lin SJ Kuo ML Hsiao HS Lee PT Lee WI Chen JY. Cytotoxic Function and Cytokine Production of Natural Killer Cells and Natural Killer T-Like Cells in Systemic Lupus Erythematosis Regulation with Interleukin-15. Mediators Inflamm (2019) 2019:4236562. 10.1155/2019/4236562 31049024 PMC6462338

[253] Mohammed RH Goyal A Bansal P. Rheumatoid Arthritis. Treasure Island (FL: StatPearls (2020).

[254] Guo Q Wang Y Xu D Nossent J Pavlos NJ Xu J. Rheumatoid arthritis: pathological mechanisms and modern pharmacologic therapies. Bone Res (2018) 6:15. 10.1038/s41413-018-0016-9 29736302 PMC5920070

[255] Romas E Gillespie MT Martin TJ. Involvement of receptor activator of NFkappaB ligand and tumor necrosis factor-alpha in bone destruction in rheumatoid arthritis. Bone (2002) 30(2):340–6. 10.1016/S8756-3282(01)00682-2 11856640

[256] Sato K Takayanagi H. Osteoclasts, rheumatoid arthritis, and osteoimmunology. Curr Opin Rheumatol (2006) 18(4):419–26. 10.1097/01.bor.0000231912.24740.a5 16763464

[257] Lange U Teichmann J Muller-Ladner U Strunk J. Increase in bone mineral density of patients with rheumatoid arthritis treated with anti-TNF-alpha antibody: a prospective open-label pilot study. Rheumatol (Oxford) (2005) 44(12):1546–8. 10.1093/rheumatology/kei082 16263785

[258] Brennan FM McInnes IB. Evidence that cytokines play a role in rheumatoid arthritis. J Clin Invest (2008) 118(11):3537–45. 10.1172/JCI36389 PMC2575731 18982160

[259] Ahern DJ Brennan FM. The role of Natural Killer cells in the pathogenesis of rheumatoid arthritis: major contributors or essential homeostatic modulators? Immunol Lett (2011) 136(2):115–21. 10.1016/j.imlet.2010.11.001 21073898

[260] Edilova MI Akram A Abdul-Sater AA. Innate immunity drives pathogenesis of rheumatoid arthritis. Biomed J (2020) 8:S2319–4170(20)30098-6. 10.1016/j.bj.2020.06.010 PMC8178572 32798211

[261] Pridgeon C Lennon GP Pazmany L Thompson RN Christmas SE Moots RJ. Natural killer cells in the synovial fluid of rheumatoid arthritis patients exhibit a CD56bright,CD94bright,CD158negative phenotype. Rheumatol (Oxford) (2003) 42(7):870–8. 10.1093/rheumatology/keg240 12730548

[262] Tak PP Kummer JA Hack CE Daha MR Smeets TJ Erkelens GW. Granzyme-positive cytotoxic cells are specifically increased in early rheumatoid synovial tissue. Arthritis Rheum (1994) 37(12):1735–43. 10.1002/art.1780371205 7986219

[263] Lo CK Lam QL Sun L Wang S Ko KH Xu H. Natural killer cell degeneration exacerbates experimental arthritis in mice via enhanced interleukin-17 production. Arthritis Rheum (2008) 58(9):2700–11. 10.1002/art.23760 18759269

[264] Soderstrom K Stein E Colmenero P Purath U Muller-Ladner U de Matos CT. Natural killer cells trigger osteoclastogenesis and bone destruction in arthritis. Proc Natl Acad Sci USA (2010) 107(29):13028–33. 10.1073/pnas.1000546107 PMC2919936 20615964

[265] Chalan P Bijzet J Kroesen BJ Boots AM Brouwer E. Altered Natural Killer Cell Subsets in Seropositive Arthralgia and Early Rheumatoid Arthritis Are Associated with Autoantibody Status. J Rheumatol (2016) 43(6):1008–16. 10.3899/jrheum.150644 27036380

[266] Aggarwal A Sharma A Bhatnagar A. Role of cytolytic impairment of natural killer and natural killer T-cell populations in rheumatoid arthritis. Clin Rheumatol (2014) 33(8):1067–78. 10.1007/s10067-014-2641-z 24797770

[267] Elemam NM Hachim MY Hannawi S Maghazachi AA. Differentially Expressed Genes of Natural Killer Cells Can Distinguish Rheumatoid Arthritis Patients from Healthy Controls. Genes (2020) 11(5):492. 10.3390/genes11050492 PMC7290970 32365786

[268] Dalbeth N Callan MF. A subset of natural killer cells is greatly expanded within inflamed joints. Arthritis Rheum (2002) 46(7):1763–72. 10.1002/art.10410 12124859

[269] Piccioli D Sbrana S Melandri E Valiante NM. Contact-dependent stimulation and inhibition of dendritic cells by natural killer cells. J Exp Med (2002) 195(3):335–41. 10.1084/jem.20010934 PMC2193592 11828008

[270] Gerosa F Baldani-Guerra B Nisii C Marchesini V Carra G Trinchieri G. Reciprocal activating interaction between natural killer cells and dendritic cells. J Exp Med (2002) 195(3):327–33. 10.1084/jem.20010938 PMC2193595 11828007

[271] Lee J Lee SH Shin N Jeong M Kim MS Kim MJ. Tumor necrosis factor-alpha enhances IL-15-induced natural killer cell differentiation. Biochem Biophys Res Commun (2009) 386(4):718–23. 10.1016/j.bbrc.2009.06.120 19559672

[272] Leipe J Grunke M Dechant C Reindl C Kerzendorf U Schulze-Koops H. Role of Th17 cells in human autoimmune arthritis. Arthritis Rheum (2010) 62(10):2876–85. 10.1002/art.27622 20583102

[273] Harrington LE Hatton RD Mangan PR Turner H Murphy TL Murphy KM. Interleukin 17-producing CD4+ effector T cells develop via a lineage distinct from the T helper type 1 and 2 lineages. Nat Immunol (2005) 6(11):1123–32. 10.1038/ni1254 16200070

[274] Takayanagi H Ogasawara K Hida S Chiba T Murata S Sato K. T-cell-mediated regulation of osteoclastogenesis by signalling cross-talk between RANKL and IFN-gamma. Nature (2000) 408(6812):600–5. 10.1038/35046102 11117749

[275] Thanapati S Ganu M Giri P Kulkarni S Sharma M Babar P. Impaired NK cell functionality and increased TNF-alpha production as biomarkers of chronic chikungunya arthritis and rheumatoid arthritis. Hum Immunol (2017) 78(4):370–4. 10.1016/j.humimm.2017.02.006 28213049

[276] Aramaki T Ida H Izumi Y Fujikawa K Huang M Arima K. A significantly impaired natural killer cell activity due to a low activity on a per-cell basis in rheumatoid arthritis. Modern Rheumatol (2009) 19(3):245–52. 10.3109/s10165-009-0160-6 19283441

[277] Ciccia F Accardo-Palumbo A Alessandro R Rizzo A Principe S Peralta S. Interleukin-22 and interleukin-22-producing NKp44+ natural killer cells in subclinical gut inflammation in ankylosing spondylitis. Arthritis Rheum (2012) 64(6):1869–78. 10.1002/art.34355 22213179

[278] Mathieu A Cauli A Fiorillo MT Sorrentino R. HLA-B27 and ankylosing spondylitis geographic distribution as the result of a genetic selection induced by malaria endemic? A review supporting the hypothesis. Autoimmun Rev (2008) 7(5):398–403. 10.1016/j.autrev.2008.03.013 18486928

[279] Braun J Bollow M Remlinger G Eggens U Rudwaleit M Distler A. Prevalence of spondylarthropathies in HLA-B27 positive and negative blood donors. Arthritis Rheum (1998) 41(1):58–67. 10.1002/1529-0131(199801)41:1<58::AID-ART8>3.0.CO;2-G 9433870

[280] Cortes A Pulit SL Leo PJ Pointon JJ Robinson PC Weisman MH. MHCcompatibility complex associations of ankylosing spondylitis are complex and involve further epistasis with ERAP1. Nat Commun (2015) 6:7146. 10.1038/ncomms8146 25994336 PMC4443427

[281] Rastall DPW Alyaquob FS O’Connell P Pepelyayeva Y Peters D Godbehere-Roosa S. Mice expressing human ERAP1 variants associated with ankylosing spondylitis have altered T-cell repertoires and NK cell functions, as well as increased in utero and perinatal mortality. Int Immunol (2017) 29(6):277–89. 10.1093/intimm/dxx035 PMC5890900 28814066

[282] Wang S Li G Ge R Duan Z Zeng Z Zhang T. Association of KIR genotype with susceptibility to HLA-B27-positive ankylosing spondylitis. Modern Rheumatol (2013) 23(3):538–41. 10.1007/s10165-012-0692-z 22744805

[283] Jiao YL Zhang BC You L Li JF Zhang J Ma CY. Polymorphisms of KIR gene and HLA-C alleles: possible association with susceptibility to HLA-B27-positive patients with ankylosing spondylitis. J Clin Immunol (2010) 30(6):840–4. 10.1007/s10875-010-9444-z 20652381

[284] Diaz-Pena R Blanco-Gelaz MA Suarez-Alvarez B Martinez-Borra J Lopez-Vazquez A Alonso-Arias R. Activating KIR genes are associated with ankylosing spondylitis in Asian populations. Hum Immunol (2008) 69(7):437–42. 10.1016/j.humimm.2008.04.012 18638658

[285] Lopez-Larrea C Blanco-Gelaz MA Torre-Alonso JC Bruges Armas J Suarez-Alvarez B Pruneda L. Contribution of KIR3DL1/3DS1 to ankylosing spondylitis in human leukocyte antigen-B27 Caucasian populations. Arthritis Res Ther (2006) 8(4):R101. 10.1186/ar1988 16805919 PMC1779409

[286] Cauli A Piga M Dessole G Porru G Floris A Vacca A. Killer-cell immunoglobulin-like receptors (KIR) and HLA-class I heavy chains in ankylosing spondylitis. Drug Dev Res (2014) 75 Suppl 1:S15–9. 10.1002/ddr.21187 25381967

[287] Shaw J Kollnberger S. New perspectives on the ligands and function of the killer cell immunoglobulin-like receptor KIR3DL2 in health and disease. Front Immunol (2012) 3:339. 10.3389/fimmu.2012.00339 23162554 PMC3499701

[288] Peruzzi M Wagtmann N Long EO. A p70 killer cell inhibitory receptor specific for several HLA-B allotypes discriminates among peptides bound to HLA-B*2705. J Exp Med (1996) 184(4):1585–90. 10.1084/jem.184.4.1585 PMC2192820 8879234

[289] Stewart-Jones GB di Gleria K Kollnberger S McMichael AJ Jones EY Bowness P. Crystal structures and KIR3DL1 recognition of three immunodominant viral peptides complexed to HLA-B*2705. Eur J Immunol (2005) 35(2):341–51. 10.1002/eji.200425724 15657948

[290] Malnati MS Peruzzi M Parker KC Biddison WE Ciccone E Moretta A. Peptide specificity in the recognition of MHC class I by natural killer cell clones. Science (1995) 267(5200):1016–8. 10.1126/science.7863326 7863326

[291] Szanto S Aleksza M Mihaly E Lakos G Szabo Z Vegvari A. Intracytoplasmic cytokine expression and T cell subset distribution in the peripheral blood of patients with ankylosing spondylitis. J Rheumatol (2008) 35(12):2372–5. 10.3899/jrheum.070839 19004035

[292] Azuz-Lieberman N Markel G Mizrahi S Gazit R Hanna J Achdout H. The involvement of NK cells in ankylosing spondylitis. Int Immunol (2005) 17(7):837–45. 10.1093/intimm/dxh270 15937057

[293] Mousavi T Poormoghim H Moradi M Tajik N Shahsavar F Soofi M. Phenotypic study of natural killer cell subsets in ankylosing spondylitis patients. Iranian J Allergy Asthma Immunol (2009) 8(4):193–8. 20404389

[294] Yang M Zhou Y Liu L Wang S Jiang J Shang Q. Decreased A20 expression on circulating CD56(bright) NK cells contributes to a worse disease status in patients with ankylosing spondylitis. Clin Exp Immunol (2019) 198(1):1–10. 10.1111/cei.13341 31206174 PMC6718289

[295] Lau MC Keith P Costello ME Bradbury LA Hollis KA Thomas R. Genetic association of ankylosing spondylitis with TBX21 influences T-bet and pro-inflammatory cytokine expression in humans and SKG mice as a model of spondyloarthritis. Ann Rheum Diseases (2017) 76(1):261–9. 10.1136/annrheumdis-2015-208677 27125523

[296] Thoen J Forre O Waalen K Pahle J. Phenotypes and spontaneous cell cytotoxicity of mononuclear cells from patients with seronegative spondyloarthropathies: ankylosing spondylitis, psoriatic arthropathy and pauciarticular juvenile chronic arthritis–analysis of mononuclear cells from peripheral blood, synovial fluid and synovial membranes. Clin Rheumatol (1988) 7(1):95–106. 10.1007/BF02284064 3261676

[297] Wendling D Racadot E Guidet M. Natural cytotoxic function and ankylosing spondylitis]. Pathologie-biologie (1989) 37(8):888–92. 2482475

[298] Cauli A Dessole G Piga M Angioni MM Pinna S Floris A. Expression analysis of HLA-E and NKG2A and NKG2C receptors points at a role for natural killer function in ankylosing spondylitis. RMD Open (2018) 4(2):e000597. 10.1136/rmdopen-2017-000597 30018803 PMC6045714

[299] Schulte-Wrede U Sorensen T Grun JR Haupl T Hirseland H Steinbrich-Zollner M. An explorative study on deep profiling of peripheral leukocytes to identify predictors for responsiveness to anti-tumour necrosis factor alpha therapies in ankylosing spondylitis: natural killer cells in focus. Arthritis Res Ther (2018) 20(1):191. 10.1186/s13075-018-1692-y 30157966 PMC6116509

[300] Sun L Xi S He G Li Z Gang X Sun C. Two to Tango: Dialogue between Adaptive and Innate Immunity in Type 1 Diabetes. J Diabetes Res (2020) 2020:4106518. 10.1155/2020/4106518 32802890 PMC7415089

[301] Nekoua MP Dechaumes A Sane F Alidjinou EK Moutairou K Yessoufou A. Enteroviral Pathogenesis of Type 1 Diabetes: The Role of Natural Killer Cells. Microorganisms (2020) 8(7):989. 10.3390/microorganisms8070989 PMC7409131 32630332

[302] Poirot L Benoist C Mathis D. Natural killer cells distinguish innocuous and destructive forms of pancreatic islet autoimmunity. Proc Natl Acad Sci USA (2004) 101(21):8102–7. 10.1073/pnas.0402065101 PMC419564 15141080

[303] Todd DJ Forsberg EM Greiner DL Mordes JP Rossini AA Bortell R. Deficiencies in gut NK cell number and function precede diabetes onset in BB rats. J Immunol (2004) 172(9):5356–62. 10.4049/jimmunol.172.9.5356 15100275

[304] Ogasawara K Hamerman JA Hsin H Chikuma S Bour-Jordan H Chen T. Impairment of NK cell function by NKG2D modulation in NOD mice. Immunity (2003) 18(1):41–51. 10.1016/S1074-7613(02)00505-8 12530974

[305] Flodstrom M Maday A Balakrishna D Cleary MM Yoshimura A Sarvetnick N. Target cell defense prevents the development of diabetes after viral infection. Nat Immunol (2002) 3(4):373–82. 10.1038/ni771 11919579

[306] Rodacki M Svoren B Butty V Besse W Laffel L Benoist C. Altered natural killer cells in type 1 diabetic patients. Diabetes (2007) 56(1):177–85. 10.2337/db06-0493 17192480

[307] Lorini R Moretta A Valtorta A d’Annunzio G Cortona L Vitali L. Cytotoxic activity in children with insulin-dependent diabetes mellitus. Diabetes Res Clin Pract (1994) 23(1):37–42. 10.1016/0168-8227(94)90125-2 7516851

[308] Negishi K Waldeck N Chandy G Buckingham B Kershnar A Fisher L. Natural killer cell and islet killer cell activities in type 1 (insulin-dependent) diabetes. Diabetologia (1986) 29(6):352–7. 10.1007/BF00903343 3527835

[309] Hussain MJ Alviggi L Millward BA Leslie RD Pyke DA Vergani D. Evidence that the reduced number of natural killer cells in type 1 (insulin-dependent) diabetes may be genetically determined. Diabetologia (1987) 30(12):907–11. 10.1007/BF00295872 3436487

[310] Nekoua MP Fachinan R Fagninou A Alidjinou EK Moutairou K Hober D. Does control of glycemia regulate immunological parameters in insulin-treated persons with type 1 diabetes? Diabetes Res Clin Pract (2019) 157:107868. 10.1016/j.diabres.2019.107868 31560963

[311] Lima JF Oliveira LMS Pereira NZ Duarte AJS Sato MN. Polyfunctional natural killer cells with a low activation profile in response to Toll-like receptor 3 activation in HIV-1-exposed seronegative subjects. Sci Rep (2017) 7(1):524. 10.1038/s41598-017-00637-3 28373665 PMC5428831

[312] Shastry A Sedimbi SK Rajalingam R Nikitina-Zake L Rumba I Wigzell H. Combination of KIR 2DL2 and HLA-C1 (Asn 80) confers susceptibility to type 1 diabetes in Latvians. Int J Immunogenet (2008) 35(6):439–46. 10.1111/j.1744-313X.2008.00804.x 19046302

[313] Shastry A Sedimbi SK Rajalingam R Rumba I Kanungo A Sanjeevi CB. Different KIRs confer susceptibility and protection to adults with latent autoimmune diabetes in Latvian and Asian Indian populations. Ann New York Acad Sci (2008) 1150:133–8. 10.1196/annals.1447.058 19120281

[314] Dunphy SE Sweeney CM Kelly G Tobin AM Kirby B Gardiner CM. Natural killer cells from psoriasis vulgaris patients have reduced levels of cytotoxicity associated degranulation and cytokine production. Clin Immunol (2017) 177:43–9. 10.1016/j.clim.2015.10.004 26477484

[315] Vicic M Kastelan M Sotosek Tokmadzic V Prpic Massari L. Systemic and Local Increase of Granulysin Expression in Cytotoxic Lymphocytes in Severe Psoriasis. Acta Dermato Venereologica (2019) 99(12):1136–42. 10.2340/00015555-3298 31449312

[316] Polese B Zhang H Thurairajah B King IL. Innate Lymphocytes in Psoriasis. Front Immunol (2020) 11:242. 10.3389/fimmu.2020.00242 32153574 PMC7047158

[317] Casciano F Pigatto PD Secchiero P Gambari R Reali E. T Cell Hierarchy in the Pathogenesis of Psoriasis and Associated Cardiovascular Comorbidities. Front Immunol (2018) 9:1390. 10.3389/fimmu.2018.01390 29971067 PMC6018171

[318] Luci C Gaudy-Marqueste C Rouzaire P Audonnet S Cognet C Hennino A. Peripheral natural killer cells exhibit qualitative and quantitative changes in patients with psoriasis and atopic dermatitis. Br J Dermatol (2012) 166(4):789–96. 10.1111/j.1365-2133.2012.10814.x 22233261

[319] Son SW Kim EO Ryu ES Kim TJ Kim JN Choi JE. Upregulation of Fas and downregulation of CD94/NKG2A inhibitory receptors on circulating natural killer cells in patients with new-onset psoriasis. Br J Dermatol (2009) 161(2):281–8. 10.1111/j.1365-2133.2009.09178.x 19438461

[320] Zeng X Chen H Gupta R Paz-Altschul O Bowcock AM Liao W. Deletion of the activating NKG2C receptor and a functional polymorphism in its ligand HLA-E in psoriasis susceptibility. Exp Dermatol (2013) 22(10):679–81. 10.1111/exd.12233 PMC3813441 24079744

[321] Patel F Marusina AI Duong C Adamopoulos IE Maverakis E. NKG2C, HLA-E and their association with psoriasis. Exp Dermatol (2013) 22(12):797–9. 10.1111/exd.12280 PMC4939276 24279916

[322] Ermis E Celik SK Solak N Genc GC Dursun A. The role of GNLY gene polymorphisms in psoriasis pathogenesis. Anais brasileiros dermatologia (2019) 94(2):198–203. 10.1590/abd1806-4841.20198188 PMC6486070 31090825

[323] Ghodke Y Joshi K Chopra A Patwardhan B. HLA and disease. Eur J Epidemiol (2005) 20(6):475–88. 10.1007/s10654-005-5081-x 16121756

[324] Agrawal S Prakash S. Significance of KIR like natural killer cell receptors in autoimmune disorders. Clin Immunol (2020) 216:108449. 10.1016/j.clim.2020.108449 32376502

[325] Enciso-Vargas M Alvarado-Ruiz L Suarez-Villanueva AS Macias-Barragan J Montoya-Buelna M Oceguera-Contreras E. Association Study between Psoriatic Arthritis and Killer Immunoglobulin-Like Receptor (KIR) Genes: A Meta-Analysis. Immunol Invest (2021) 50(2–3):152–63. 10.1080/08820139.2020.1713145 31957514

[326] Luszczek W Manczak M Cislo M Nockowski P Wisniewski A Jasek M. Gene for the activating natural killer cell receptor, KIR2DS1, is associated with susceptibility to psoriasis vulgaris. Hum Immunol (2004) 65(7):758–66. 10.1016/j.humimm.2004.05.008 15310528

[327] Macias-Barragan J Montoya-Buelna M Enciso-Vargas M Alvarado-Ruiz L Oceguera-Contreras E Guerra-Renteria AS. Assessment of the Relationship between Clinical Variants of Psoriasis and Killer Immunoglobulin-like Receptor (KIR) Genes: A Systematic Review with Meta-analysis. Immunol Invest (2020) 1–16. 10.1080/08820139.2020.1840582 33115277

[328] Chen L Tsai TF. HLA-Cw6 and psoriasis. Br J Dermatol (2018) 178(4):854–62. 10.1111/bjd.16083 29072309

[329] Holm SJ Sakuraba K Mallbris L Wolk K Stahle M Sanchez FO. Distinct HLA-C/KIR genotype profile associates with guttate psoriasis. J Invest Dermatol (2005) 125(4):721–30. 10.1111/j.0022-202X.2005.23879.x 16185272

[330] Kulkarni S Martin MP Carrington M. The Yin and Yang of HLA and KIR in human disease. Semin Immunol (2008) 20(6):343–52. 10.1016/j.smim.2008.06.003 PMC3501819 18635379

[331] McGonagle D Aydin SZ Gul A Mahr A Direskeneli H. ‘MHC-I-opathy’-unified concept for spondyloarthritis and Behcet disease. Nat Rev Rheumatol (2015) 11(12):731–40. 10.1038/nrrheum.2015.147 26526644

[332] Prakash S Alam S Bharadwaj U Aggarwal A Mishra RN Agrawal S. Associations of killer cell immunoglobulin like receptors with rheumatoid arthritis among North Indian population. Hum Immunol (2014) 75(8):802–7. 10.1016/j.humimm.2014.05.014 24912006

[333] Williams F Meenagh A Sleator C Cook D Fernandez-Vina M Bowcock AM. Activating killer cell immunoglobulin-like receptor gene KIR2DS1 is associated with psoriatic arthritis. Hum Immunol (2005) 66(7):836–41. 10.1016/j.humimm.2005.04.005 16112031

[334] Eyerci N Balkan E Akdeniz N Keles S. Association of MICA Alleles and Human Leukocyte Antigen B in Turkish Patients Diagnosed With Behcet’s Disease. Arch Rheumatol (2018) 33(3):352–7. 10.5606/ArchRheumatol.2018.6704 PMC6328215 30632534

[335] Gambelunghe G Brozzetti A Ghaderi M Candeloro P Tortoioli C Falorni A. MICA gene polymorphism in the pathogenesis of type 1 diabetes. Ann New York Acad Sci (2007) 1110:92–8. 10.1196/annals.1423.011 17911424

[336] Groh V Bruhl A El-Gabalawy H Nelson JL Spies T. Stimulation of T cell autoreactivity by anomalous expression of NKG2D and its MIC ligands in rheumatoid arthritis. Proc Natl Acad Sci USA (2003) 100(16):9452–7. 10.1073/pnas.1632807100 PMC170939 12878725

[337] Alter G Martin MP Teigen N Carr WH Suscovich TJ Schneidewind A. Differential natural killer cell-mediated inhibition of HIV-1 replication based on distinct KIR/HLA subtypes. J Exp Med (2007) 204(12):3027–36. 10.1084/jem.20070695 PMC2118524 18025129

[338] Petrushkin H Norman PJ Lougee E Parham P Wallace GR Stanford MR. KIR3DL1/S1 Allotypes Contribute Differentially to the Development of Behcet Disease. J Immunol (2019) 203(6):1629–35. 10.4049/jimmunol.1801178 PMC6731450 31405953

[339] Conigliaro P Scrivo R Valesini G Perricone R. Emerging role for NK cells in the pathogenesis of inflammatory arthropathies. Autoimmun Rev (2011) 10(10):577–81. 10.1016/j.autrev.2011.04.017 21536152

